# Ecological plasticity in the gastrointestinal microbiomes of Ethiopian *Chlorocebus* monkeys

**DOI:** 10.1038/s41598-017-18435-2

**Published:** 2018-01-08

**Authors:** Pål Trosvik, Eli K. Rueness, Eric J. de Muinck, Amera Moges, Addisu Mekonnen

**Affiliations:** 1 Centre for Ecological and Evolutionary Synthesis (CEES), Department of Biosciences, University of Oslo, Oslo, Norway; 2 Department of Biology, Bahir Dar University, Bahir Dar, Ethiopia; 3 Department of Zoological Sciences, Addis Ababa University, Addis Ababa, Ethiopia

## Abstract

Human activities can cause habitat degradation that may alter the types and quality of available food resources and thus influence the microbiomes of wild animal populations. Furthermore, seasonal shifts in food availability may cause adaptive responses in the gut microbiome to meet the need for different metabolic capabilities. Here, we demonstrate local-scale population structure in the gastrointestinal microbiotas of *Chlorocebus* monkeys, in southern Ethiopia, in response to varying degrees of human encroachment. We further provide evidence of adaptation to ecological conditions associated with the dry and wet seasons, and show seasonal effects to be more pronounced in areas with limited human activity. Finally, we report species-level microbiota differences between the endemic Ethiopian Bale monkey, an ecological specialist, and generalist *Chlorocebus* species from the same geographical region.

## Introduction

Much recent research has documented the important roles that the microbes inhabiting the gastrointestinal (GI) system play in host physiology^1^. Several studies have documented the role of diet in shaping the GI microbiota of humans^2,3^ and other mammals^4,5^. A recent study even demonstrated humanization of the GI microbiota in zoo monkeys, due to reduced intake of dietary fiber in the captive primates, indicating a high degree of plasticity in response to diet^6^.

**Figure 1:**
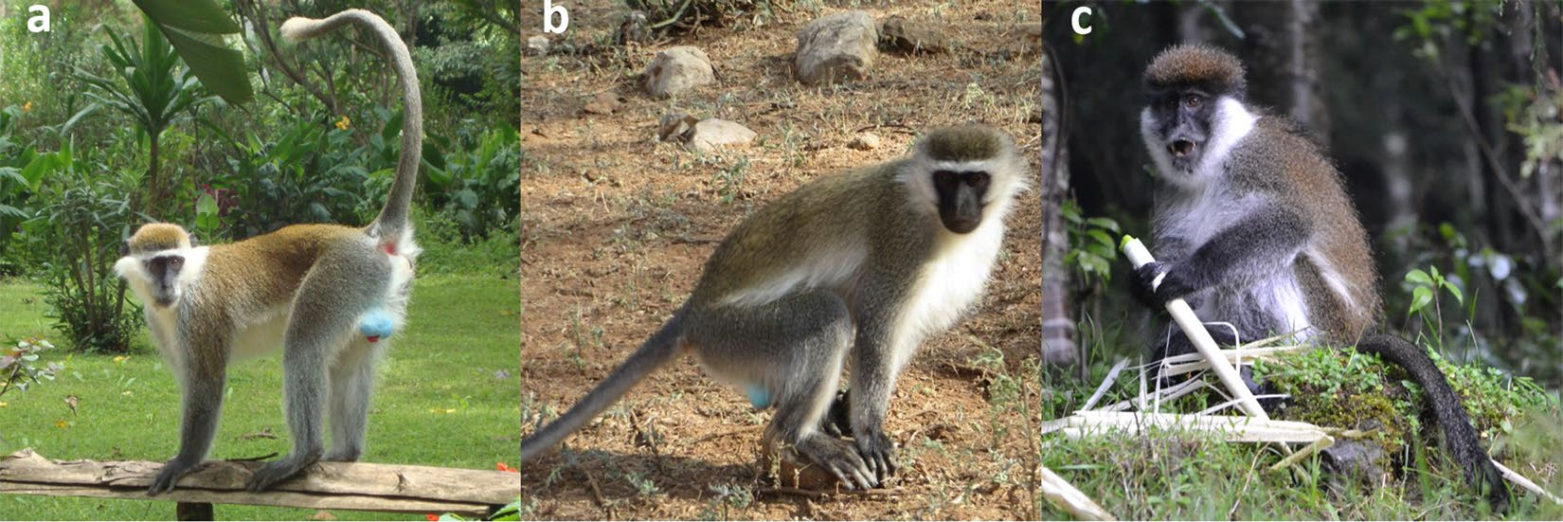
Ethiopian *Chlorocebus* monkeys. (**a**) Grivet, (**b**) vervet and (**c**) Bale monkey.

**Figure 2:**
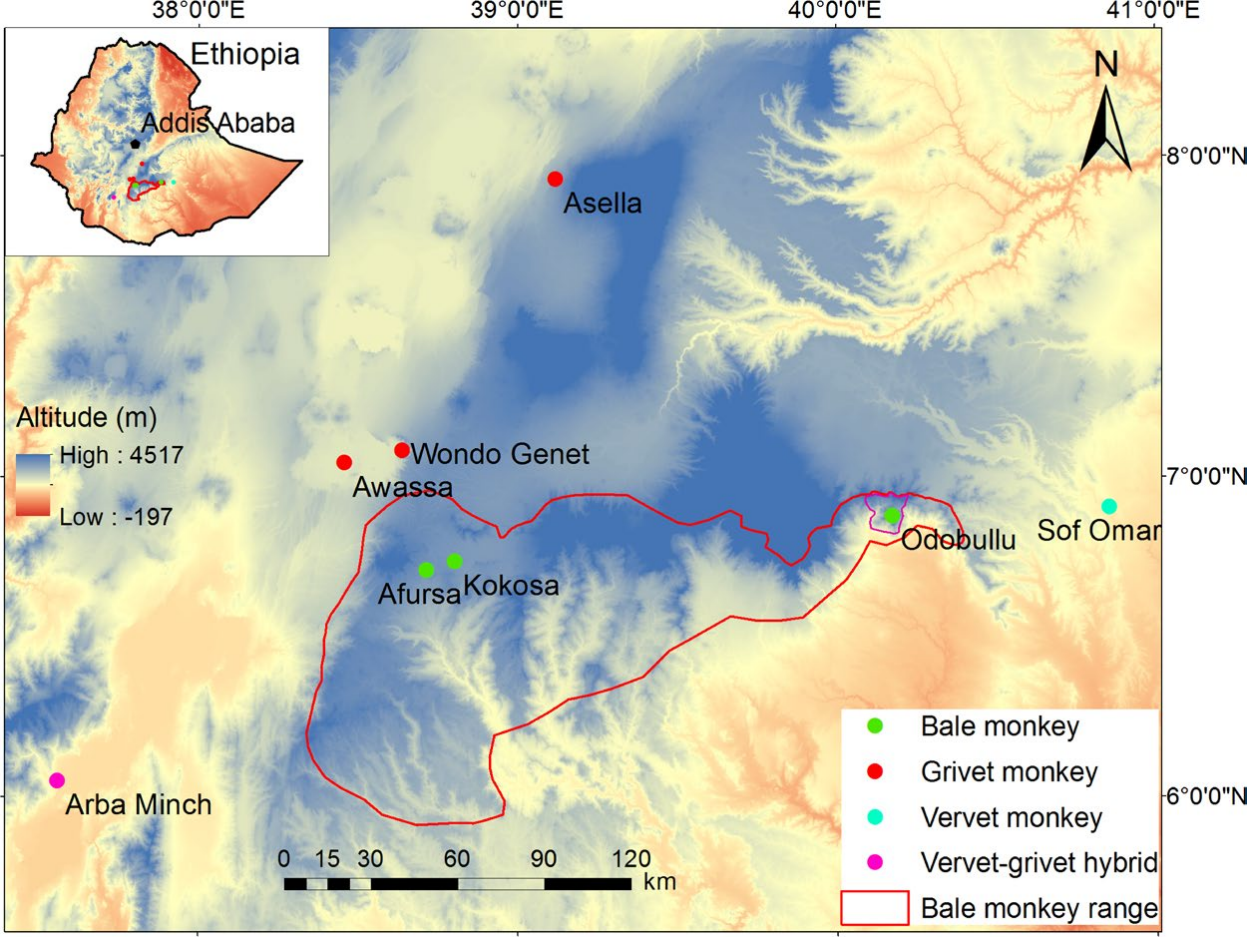
Map of the sampling area. The red outline shows the habitat range of the Bale monkeys. The map was generated using a 30 meter digital elevation model in ArcGIS 10.4 (www.arcgis.com).

The *Chlorocebus* is a genus of non-human primates widespread in sub-Saharan Africa that includes grivets (*C. aethiops*, Fig. 1a), vervets (*C. pygerythrus*, Fig. 1b) and Bale monkeys (*C. djamdjamensis*, Fig. 1c). Grivets are distributed across northern Ethiopia and surrounding areas, while vervets are found across large parts of southeast Africa. The habitats of vervets and grivets overlap in southern Ethiopia where interbreeding is believed to occur^7^ (Fig. 2). The Bale monkey is endemic to the Bale Mountains of Southern Ethiopia, and is thus parapatric with grivets and vervets^8^. Grivets and vervets are considered dietary generalists, feeding on a variety of fruits, seeds and pods^9–11^. Bale monkeys are considered dietary specialists who, along with the golden monkeys (*Cercopithecus kandti*) of Uganda^12^ and the lemurs of the genus *Hapalemur* (bamboo lemurs) of Madagaskar^13^, are the only primate species adapted to eating mostly bamboo^14^. Due to a large increase in human settlements, much of the original bamboo forest that forms the natural Bale monkey habitat has been cut down. This habitat fragmentation has resulted in populations becoming isolated in habitats with extensive human influence^8^. Two such areas are Afursa and Kokosa (Fig. 2). In Afursa (hereby referred to as NBF = no bamboo forest) bamboo growth is almost completely eradicated, while Kokosa (hereby referred to as DBF = degraded bamboo forest) consists of spread out patches of bamboo growth^15^. Bale monkeys living here have largely abandoned a bamboo-dominated diet. In contrast, Odobullu (hereby referred to as CBF = continuous bamboo forest; Fig. 2) covers a large area of predominantly dense bamboo growth, representing a natural habitat.

In this study we use 16S rRNA gene amplicon sequencing to compare GI microbiotas in Bale monkeys from the three study sites, addressing three main hypotheses. First, ecological differences on a local spatial scale, driven by habitat degradation, may cause GI microbiota differences among Bale monkeys at different sampling sites. Secondly, seasonal differences in food availability may cause GI microbiota variation within Bale monkey sampling sites. Lastly, ecological specializations in Bale monkeys may cause their GI microbiota to differ from the more generalist *Chlorocebus* species. We also make an effort to interpret our microbiota data using chloroplast 16S rRNA gene sequence reads, produced as a bi-product of amplicon sequencing, as a potential source of dietary information.

## Results

### GI microbiota composition of the Bale monkeys in the three study sites

**Figure 3:**
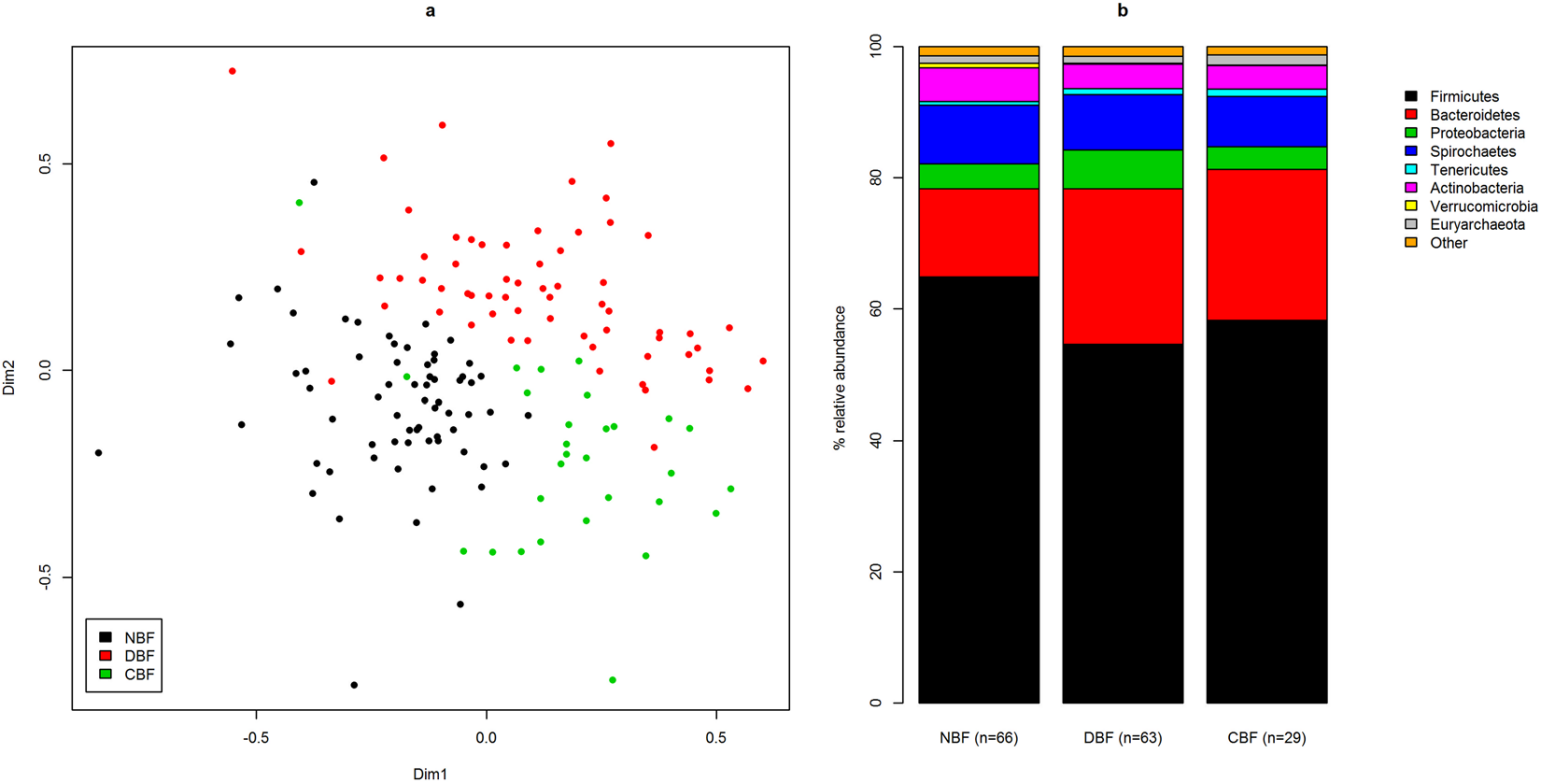
NMDS and phylum level relative abundances for 158 Bale monkey samples from the populations NBF, DBF and CBF. (**a**) The NMDS plot shows the two main dimensions of variation, with dots colour coded according to sampling site. (**b**) Relative abundances are shown for the top eight phyla.

The three Bale monkey populations clustered according to study site (p < 0.001, ANOSIM). The main dimension of variation distinguished between NBF, on the one hand, and DBF and CBF on the other (Fig. 3a). The second dimension mostly distinguished between the DBF and CBF populations. On the phylum level, differences between the three populations were mainly related to the Firmicutes vs. Bacteroidetes balance (Fig. 3b). The population mean relative abundance of Firmicutes was highest in in NBF (64.9%), followed by CBF (58.2%), and DBF (54.7%) (p < 0.001 for all comparisons, Wilcoxon rank sum test). Conversely, Bacteroidetes relative abundances were lower in NBF (13.4%) than in CBF (23.0%) or DBF (23.7%) (p < 0.001 for both comparisons). We did not observe significant differences in either Firmicutes or Bacteroidetes abundances between the DBF and CBF populations. Other significant phylum-level differences between populations were elevated abundances of Spirochaetes in DBF relative to both of the other populations (p = 0.003 and 0.021 for comparison with NBF and CBF, respectively), higher levels of Euryarchaeota (methanogenic archaea) in CBF relative to NBF and DBF (p < 0.001 and p = 0.01, respectively), and higher levels of Tenericutes in NBF (p < 0.001 and p = 0.004 compared with DBF and CBF, respectively). A relatively rare phylum, Lentisphaera, was enriched almost nine fold in NBF (0.68%) compared with CBF (0.08%) (p < 0.001) and four fold relative to DBF (0.17%) (p < 0.001).

We also carried out exact tests for enrichments of specific OTUs among the three populations. Using a strict significance cut-off of both p-value and false discovery rate (FDR) ≤0.01 we found 83 OTUs with differential occurrence between NBF and DBF, 54 between NBF and CBF and 37 between DBF and CBF (Supplementary Tables 1–3). There was extensive sharing of enriched OTUs when comparing one of the populations with the two others. In particular, when comparing NBF with either DBF or CBF, the top two OTUs discriminating between the populations were enriched more than a hundredfold in both of the two latter populations. Both of these OTUs were classified with low confidence, one to the genus *Parabacteroides* (OTU56, phylum Bacteroidetes) and the other to the genus *Asteroleplasma* (OTU26, phylum Tenericutes). A total of 38 OTUs showed significant differential occurrence in NBF relative to both DBF and CBF, and out of these OTUs 36 co-varied in the sense that they were either enriched or depleted in both DBF and CBF. We saw a similar trend when looking at OTUs with significant differential occurrence in DBF relative to NBF and CBF, with 17 out of 22 shared OTUs co-varying. For OTUs with shared differential occurrence in CBF relative to NBF and DBF, 16 out of 17 co-varied. Thus, there was a trend that an OTU that distinguished a first population from a second also distinguished the first population from the third.

We did find some effects of sampling site on the observed OTU diversity (Supplementary Fig. 1), when considering all the bale monkey samples together regardless of season. DBF had consistently lower diversity as measured by Shannon entropy, OTU richness and the Chao1 diversity estimator. However, results from significance testing were somewhat inconsistent (Supplementary Table 4).

### Seasonal plasticity

**Figure 4:**
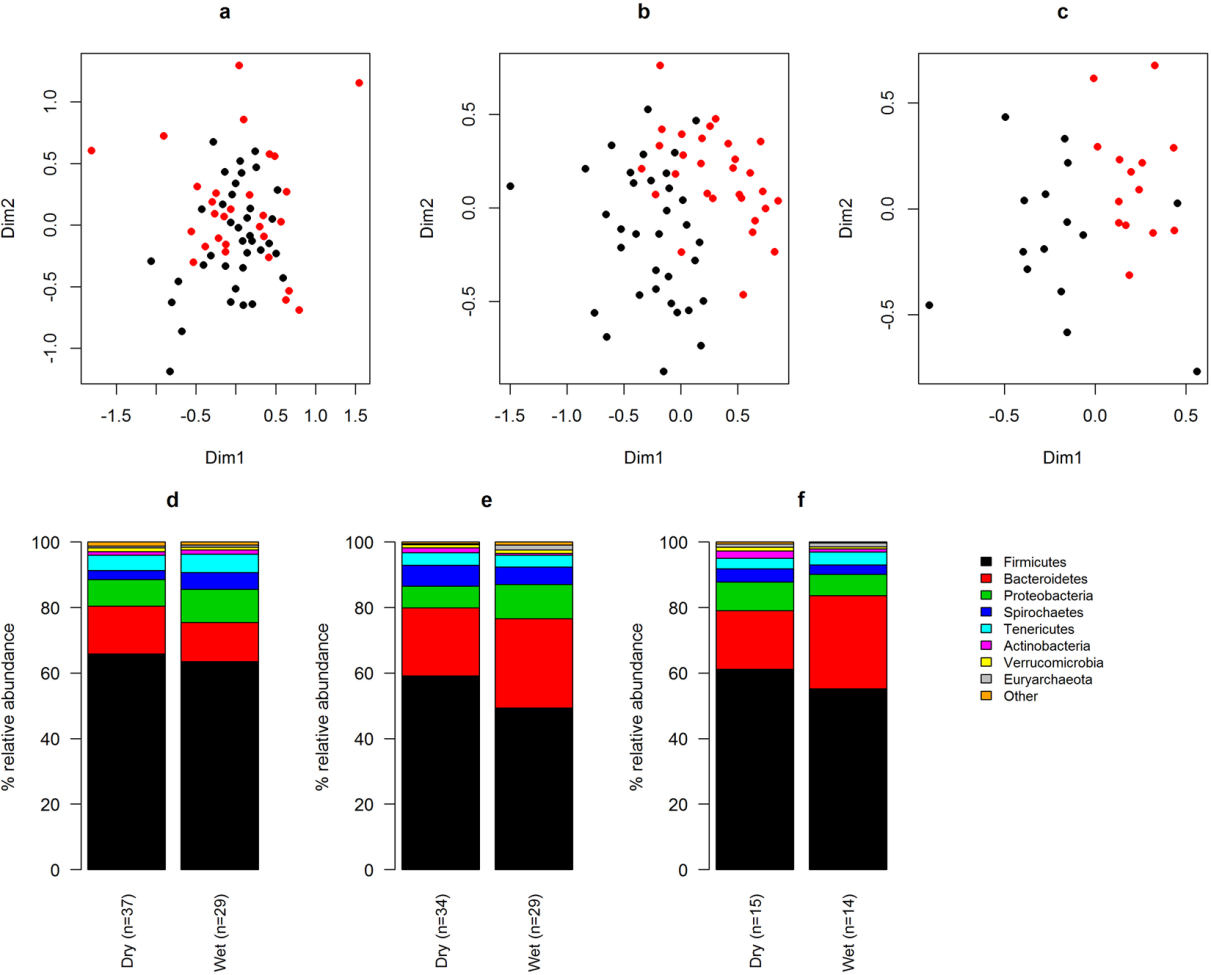
NMDS and phylum level relative abundances in the Bale monkey samples with samples split into those collected during the dry and wet season. The panels show the populations in NBF (**a** and **d**), DBF (**b** and **e**) and CBF (**c** and **f**). The NMDS plots (**a**–**c**) shows the two main dimensions of variation, with dots colour coded according to season (black = dry, red = wet). Relative abundances (**d**–**f**) are shown for the top eight phyla.

Fecal samples from Bale monkeys were collected between October and June, with October through February being a dry season and March through June a wet season. In order to look for seasonal effects in the three Bale monkey populations we compared the microbiota composition of samples collected during these two periods. In the NBF population we observed significant differences in the microbiotas dependent on season (Fig. 4a; p < 0.001, ANOSIM), with the distinction between the dry and wet season explaining 4.3% of inter-sample variation (PERMANOVA). For the DBF and CBF populations we saw stronger seasonal effects (Fig. 4b,c; p < 0.001 for both tests) with season explaining 9.1% and 10.6% of inter-sample variation, respectively.

At the phylum level, in NBF there was a marginally significant (p = 0.048, Wilcoxon rank sum test) decrease in Bacteroidetes abundances from the dry to the wet season (Fig. 4d), while Spirochaetes and Actinobacteria increased significantly (p = 0.010 and 0.021, respectively). In both DBF (Fig. 4e) and CBF (Fig. 4f), Firmicutes decreased in the wet relative to the dry season (p = 0.002 and 0.056 in DBF and CBF, respectively), with a concurrent increase in Bacteroidetes (p = 0.011 and 0.006). In DBF there were significant increases in Proteobacteria and Euryachaeota (p < 0.001 in both cases) and decreases in Actinobacteria and Fibrobacteres (p < 0.001 and p = 0.001), from the dry to the wet season. In CBF there were significant decreases in Spirochaetes and Actinobacteria (p = 0.029 and 0.026) as well as a marginally significant decrease in Verrucomicrobia (p = 0.057).

On the 97% OTU level, bacterial groups with significant enrichment in one season relative to the other were found in all three Bale monkey populations, with 18, 47 and 14 OTUs observed with a p-value and FDR of less than 0.01 in NBF, DBF and CBF, respectively (Supplementary Tables 5–7). No OTU with differential seasonal abundance was observed in all three populations, but five of the same OTUs were found to have significant seasonal enrichment in both NBF and DBF, one (tentatively classified to the Proteobacterial genus *Vampirovibrio*, a bacterial predator) during the wet season and four (all Firmicutes) during the dry season. Also, a single OTU (a Bacteroidetes tentatively classified as Barnsiella) was enriched in the wet season in both DBF and CBF. Thus, it appears that, in spite of a few communalities, the seasonal response on the 97% OTU level is particular to the area in which the monkeys live. We did not observe significant differences in OTU diversity between seasons in any of the Bale monkey populations, except for a marginally significant (p = 0.049, unpaired t-test) increase in the Chao1 diversity estimator in the wet relative to the dry season in CBF (Supplementary Fig. 2).

### Comparison between specialist and generalist *Chlorocebus* species

**Figure 5:**
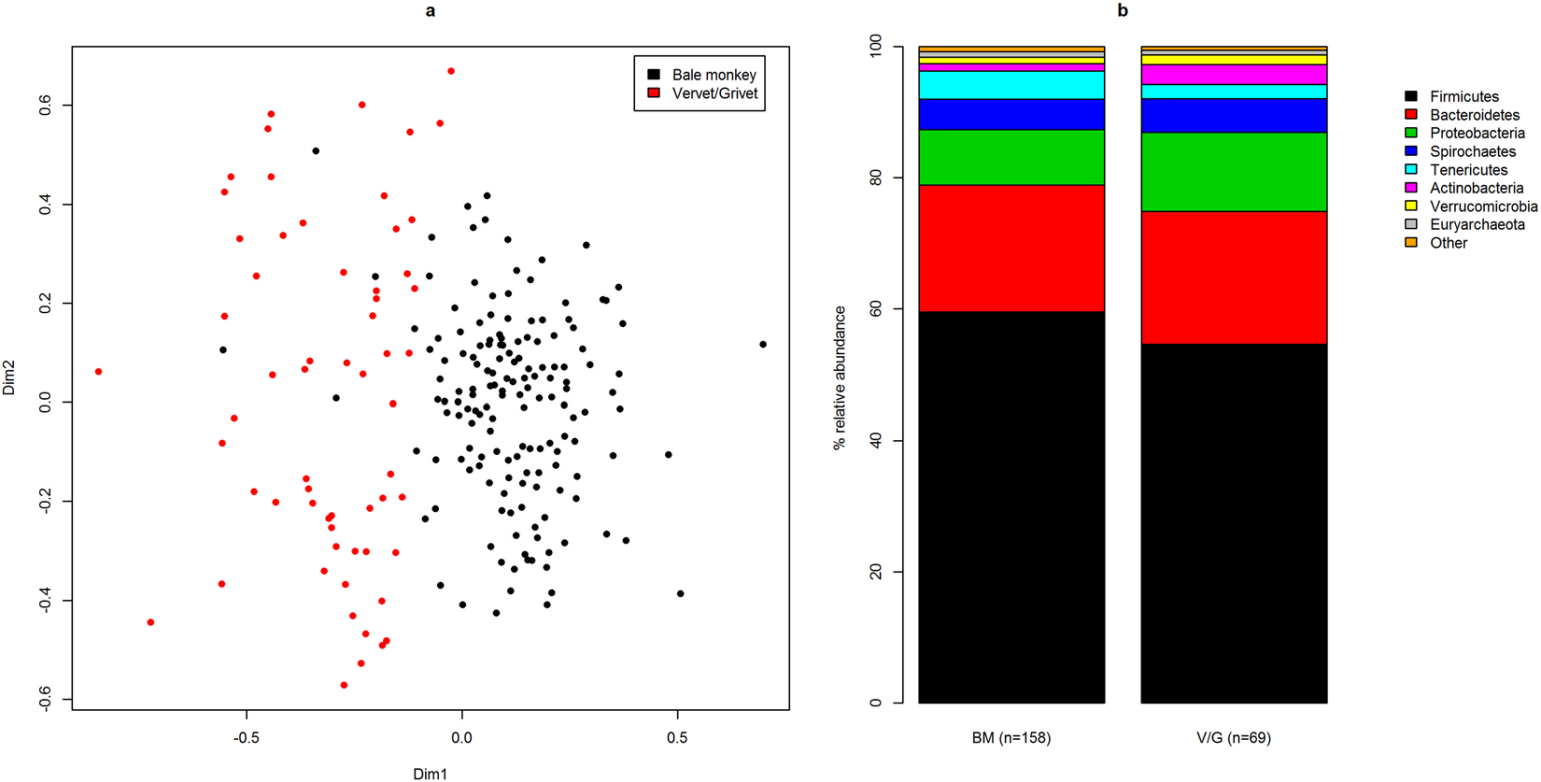
NMDS and phylum level relative abundances for the three Bale monkey populations (BM) combined and the five vervet and grivet populations (V/G) combined. (**a**) The NMDS plot shows the two main dimensions of variation, with dots colour coded according to species (top right corner). (**b**) Relative abundances are shown for the top eight phyla.

In order to look for differences in the GI microbiota between Ethiopian *Chlorocebus* species, we compared the three Bale monkey populations to the five populations of vervets and grivets. Sixty-four (4.9% of total) OTUs were found in all 219 samples from Bale, grivet and vervet monkeys. 334 OTUs (25.7% of total) were common to at least 90% of samples, suggesting a core microbiota in *Chlorocebus* monkeys. There was a highly significant separation between the Bale monkeys and the combined vervet and grivet populations (Fig. 5a; p < 0.001 ANOSIM), with the first NMDS dimension providing the main distinction. By comparing sample scores along the main NMDS dimension (Supplementary Fig. 3) the DBF population of Bale monkeys appeared more similar to vervets and grivets than Bale monkeys from NBF and CBF (mean score 0.05, p < 0.001 for both comparisons, Wilcoxon rank sum test), while the two latter populations did not differ significantly from one another (mean scores 0.15 and 0.18, respectively). We did not observe significant differences when comparing all three grivet populations to the one pure vervet population (Supplementary Fig. 4). However, we observed a strong geographical effect among the five vervet and grivet populations (p < 0.001, ANOSIM), with sampling site explaining 27.2% of inter-sample variation (PERMANOVA). At the southernmost site, Arba Minch, extensive hybridization is thought to occur between vervets and grivets^7^. Samples from this site clustered poorly (Supplementary Fig. 4), suggesting that interbreeding may affect the microbiota. Looking at all eight *Chlorocebus* populations, we did not find any evidence to suggest that geographical proximity causes GI microbiotas to be more similar (rho = −0.08, p = 0.70, Spearman’s rank correlation test).

On the phylum level there were extensive differences between Bale monkeys and vervets/grivets (Fig. 5b). Firmicutes, Tenericutes and Fibrobacteres were more abundant in the Bale monkeys (p = 0.001 for Firmicutes and p < 0.001 for the two others), while Proteobacteria, Verrucomicrobia and Actinobacteria were more abundant in vervets and grivets (p = 0.006 and 0.032 and p < 0.001, respectively). 94 of the most abundant OTUs were significantly (p-value and FDR ≤ 0.01) enriched in either Bale monkeys or vervets/grivets (Supplementary Table 8). The majority (66.0%) of enriched OTUs were classified as Firmicutes, but the three most highly enriched OTUs were all Bacteroidetes, one tentatively classified to the genus Barnsiella and two as Prevotella. These three OTUs were enriched more than a hundredfold in the vervet and grivet populations. The GI microbiota of the Bale monkey populations was more diverse than that of the vervets and grivets (Supplementary Fig. 5), both in terms of Shannon entropy (p < 0.001, unpaired t-test), OTU richness (p = 0.011) and the Chao1 diversity estimator, although the difference in Chao1 diversity was not significant.

### Chloroplasts’ composition: implication for geographical and seasonal differentiation

The NBF population had the highest number of chloroplast reads (8.7% of total) followed by CBF (5.5%) and DBF (2.2%), while chloroplast sequences were relatively rare in the vervet and grivet populations (Supplementary Fig. 6). Thus, further analyses focuses only on the three Bale monkey populations. It should be noted that chloroplast 16S rRNA sequences do not provide good resolution as a taxonomic marker in plants, and this should be taken into account when interpreting the following results.

**Figure 6:**
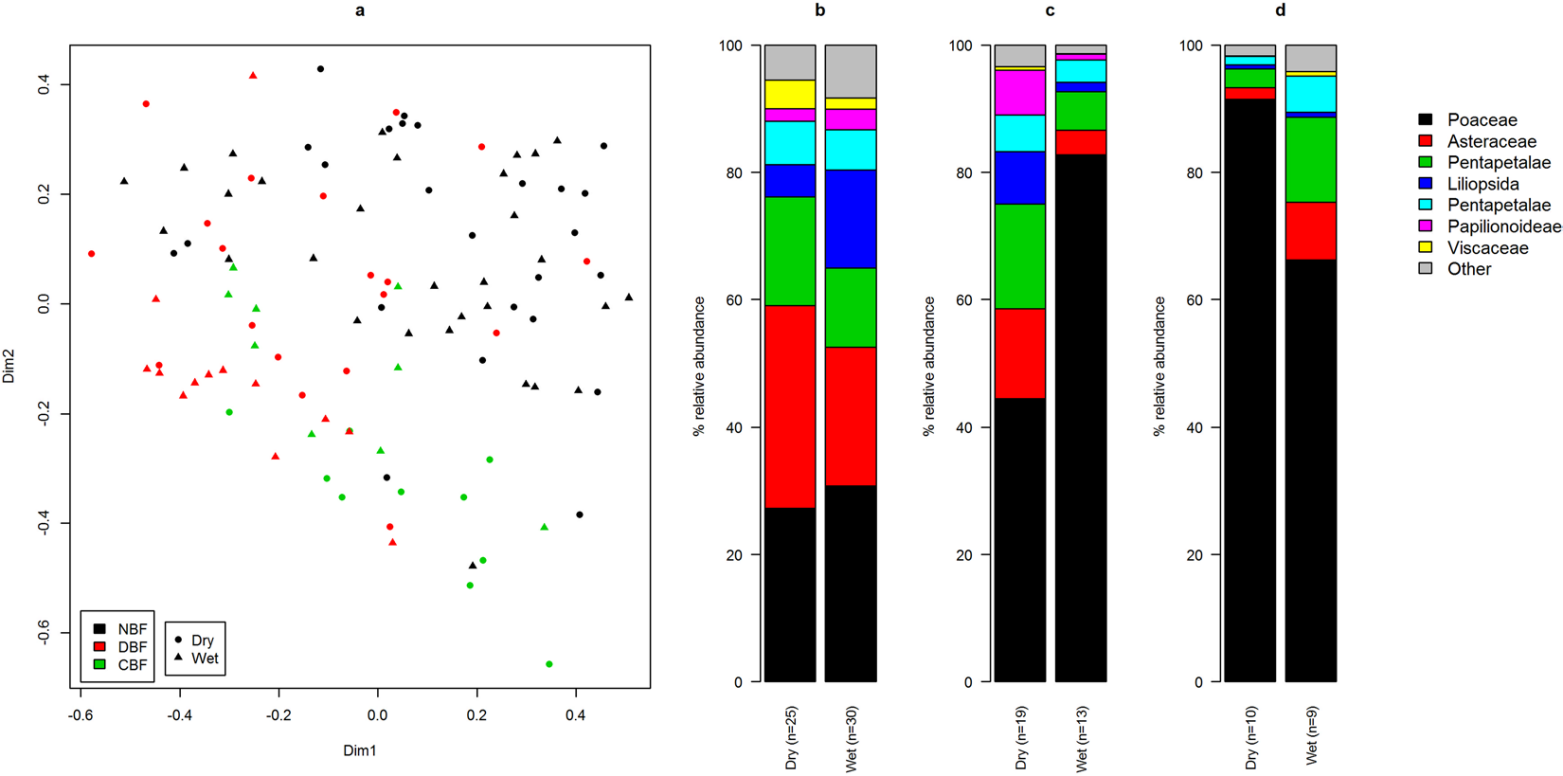
NMDS and relative abundances of OTUs classified as chloroplasts in 106 Bale monkey samples. (**a**) The NMDS plot shows the two main dimensions of variation, with plotted symbols colour coded according to sampling site (NBF = no bamboo forest, DBF = degraded bamboo forest, CBF = continuous bamboo forest) and shaped according to season. (b-d) Relative abundances of the seven top OTUs classified as chloroplasts. Samples from NBF (**b**), DBF (**c)** and CBF (**d**) are split into those collected during the dry and wet seasons. The colour key and classification shown on the right hand side give only the high level taxonomy (family and up).

There was a clear geographical signature in the chloroplast sequences (Fig. 6a; p < 0.001, ANOSIM), with sampling site explaining 18% of between-sample variation (PERMANOVA). The chloroplast sequence data suggested that the NBF population had a much more diverse diet, as measured by Shannon entropy (Supplementary Fig. 7; p < 0.001 for both comparisons, unpaired t-test). Diversity in DBF was higher than in CBF with marginal significance (p = 0.042). We found 9 bacterial OTUs that had a significant (p ≤ 0.05, Spearman’s rank correlation tests) positive correlation with the relative abundance of sequences putatively classified as Poaceae (grass), and 7 of these OTUs were classified to the order Clostridia (Supplementary Table 9).

While we did not observe significant seasonal effects of chloroplast sequence composition in the NBF population (Fig. 6b), we did see significant effects in DBF and CBF (Fig. 6c,d; p = 0.023 and 0.021, respectively; ANOSIM). We observed opposite trends at the two latter sites such that in DBF the chloroplast composition during the dry season was similar to that of NBF, with consumption of grassy plants (Poaceae) nearly halved relative to the wet season (44.4% vs. 82.8%; p < 0.001, Wilcoxon rank sum test). In CBF, on the other hand, grass consumption was greatly reduced in the wet relative to the dry season (66.2% vs. 91.5%, p < 0.001). Recovery of chloroplast sequences was higher in samples collected during the dry relative to the wet season in DBF and CBF (Supplementary Fig. 8).

## Discussion

It is well established that the GI microbiota is instrumental to digestive function in animals, and that diet and microbiota composition are intimately linked. This is especially true for herbivorous creatures that rely on a high proportion of recalcitrant carbohydrates for nutrition, such as ruminants^16,17^ and many primate species^18^. There is also documentation that fragmentation and other kinds of habitat disturbance can cause dietary shifts in animal populations relative to those residing in pristine habitats^19,20^. Indeed, two previous studies of primates concluded that habitat degradation can significantly affect the GI microbiota^21,22^.

CBF represents a uniform and continuous environment with bamboo, a member of the family Poaceae, accounting for more than 86% of the total vegetation^15^. The Bale monkey population in this area relies on a single species of bamboo (*Arundinaria alpina*) for most of their dietary intake (Mekonnen *et al*., in review). Thus, these animals are considered ecological specialists, and until recently they were thought to exist only in the relatively undisturbed bamboo forests of the Bale Mountains. However, the discovery of populations living in small and isolated forest fragments in human-dominated areas suggests that they are more ecologically flexible than previously thought^8^.

In DBF bamboo has recently been found to account for ~40% of the vegetation, while the corresponding percentage in NBF was only 1.6%. Thus, although both forest fragments are subject to high anthropogenic influence, DBF is intermediate in terms of bamboo access (Mekonnen *et al*., in review). Access to bamboo is in rough correspondence with the number of chloroplast reads putatively classified as Poaceae recovered from the three sampling sites (Fig. 6, Supplementary Fig. 7). In CBF, averaged over both seasons, Poaceae constituted about 80% of the total chloroplast sequences. This corresponds very well with the empirically observed rates at which monkeys there feed on bamboo (Mekonnen *et al*., in review). In DBF, monkeys spend ~30% of their foraging time feeding on bamboo, while sequences matching Poaceae were ~60% of total chloroplasts. This population is known to spend large amounts of time in human agricultural areas^15^, feeding on grasses and herbs, as well as fruit and other crops, i.e. plant parts without chloroplasts. This may help explain the relatively low number of chloroplast reads recovered from these monkeys, as well as the discrepancy between observed feeding rates and recovered grass sequences. In NBF the amount of Poaceae sequences was greatly reduced relative to the two other sites. The main grass species there is the so called smelly grass (*Bothriochloa radicans*), which takes up ~15% of the Bale monkeys’ foraging time, and they have adopted a range of other flowering plants as major components of their green diet, including shrubs, herbs and trees.

We observed clear differentiation of the Bale monkey GI microbiota among the three habitat types (Fig. 3a). We found that monkeys from the two areas with better access to bamboo had more similar GI microbiotas compared to the low bamboo site, both having lower levels of Firmicutes and much higher levels of Bacteroidetes (Fig. 3b). We also found that that several OTUs were highly enriched in the high and intermediate bamboo sites relative to the low bamboo site (Supplementary Tables 1–3). Two OTUs, putatively classified to the genera *Parabacteroides* and *Asteroleplasma*, were particularly highly enriched in monkeys in CBF and DBF. *Parabacteriodes* is a typical component of mammalian GI microbiotas, including those of humans and chimpanzees^23^. *Asteroleplasma* is a poorly characterized genus within the Tenericutes, but two recent studies have identified these bacteria as part of the rumen microbiota^24,25^, suggesting that they may play a role in cellulose degradation. The taxonomic assignment of these OTUs should be interpreted with caution as both were classified with low confidence. The DBF population had consistently lower GI microbiota diversity than the two other populations, although the significance of differences between populations was inconsistent. We do not have a good explanation for this result. It is possible that it is related to a larger component of fruit, which is more easily digested than green plant parts, in the diet of the DBF population. It is known that herbivores have more diverse GI microbiotas than omnivores^4^, and this interpretation is supported by the number of recovered chloroplast sequences from the three Bale monkey populations (Supplementary Fig. 6).

Looking to the human GI microbiome, the most studied microbiome of any primate, it has been found that, although it is highly variable between individuals^26^, it is remarkably stable over time^27^. However, seasonal population level variation in the GI microbiota has been reported both in humans^28^ and non-human primates^29,30^, as well as wild wood mice^31^ and giant pandas^32^. In Bale monkeys we observed seasonal plasticity of the GI microbiotas of all three populations (Fig. 4a–c), with elevated levels of Firmicutes and lower levels of Bacteroidetes during the dry season in each of the populations (Fig. 4d–f). These effects are presumably related to the diversity of ingested food items and the relative amounts of bamboo leaves and other green grasses in their diet. A study of lowland and mountain gorillas similarly found that seasonal variation of the GI microbiota corresponded with fruit availability^33^. It is noteworthy that the seasonal signal was weakest in the NBF population (Fig. 4a and d), suggesting that these monkeys are becoming adapted to a food supply that is less dependent on seasonal availability, an observation that is supported by the chloroplast data (Fig. 6b). The seasonal effect was strongest in CBF (Fig. 4c and f), in accordance with a recent study of howler monkeys living in habitats with different degrees of disturbance which also found more pronounced seasonal effects on the GI microbiota of animals living in habitats with little human influence^34^. In the chloroplast data, we saw opposing seasonal trends in DBF and CBF, with the relative amount of Poaceae sequences increasing in the wet season in DBF while decreasing in CBF (Fig. 6c and d). These patterns may be explained by differences in the ecology at the two sites. In CBF, during the wet season, the Bale monkeys supplement their bamboo leaf intake with fresh shoots. The shoots do not contain chloroplasts, and thus are not observable in our data. In DBF the wet season brings a bloom in the abundance of many grass and herb species that are favoured by the monkeys, thus shifting their diets away from the flowering plants consumed during the dry season. Surprisingly, we observed only marginal effects of season on GI microbiota diversity within sampling sites. While it is fully possible that the Bale monkey GI microbiota does not respond strongly to seasonal ecological changes in terms of OTU diversity, it is also possible that sample sizes become too small to detect significant differences when splitting the data from each site into samples collected during the dry and wet season. This may be particularly important in the CBF population, where the sample size is smallest and we would expect the strongest seasonal effect.

In addition to feeding ecology, phylogeny has been found to be an important determinant of GI microbiome structure^4^, and differences have been found even among closely related non-human primates^35^. In our data we saw clear signs of differentiation between Bale monkeys and vervets/grivets (Fig. 5a). The vervet and grivet populations studied here were all from habitats with a high degree of human influence, and both of these species are well known ecological generalists. The Bale monkey populations of NBF and DBF have adapted to more generalist lifestyles than their conspecifics in CBF, and in particular the GI microbiota of the DBF population was more similar to those of vervets and grivets. However, the main contrast was between Bale monkeys on the one hand and vervets and grivets on the other, suggesting that in this case the phylogenetic signal overpowers the signal caused by the degree of dietary generalism. It is noteworthy that, although rare in all populations, the phylum Fibrobacteres was significantly enriched in Bale monkeys, by a factor of 3.4, relative to vervets and grivets. This phylum is mainly associated with cellulose digestion in ruminants^36^, and enrichment of these bacteria in Bale monkeys may signify adaptation to a high-fiber diet relative to the other *Chlorocebus* species. We also found that OTU diversity was elevated in Bale moneys relative to vervets and grivets (Supplementary Fig. 5). A previous study found that, in mammals, herbivores harbour more diverse GI microbiotas than omnivores^4^, while a study of two human populations found higher diversity in the one with a higher fiber diet^2^. Thus, our result may reflect an adaptation in Bale monkeys to a diet richer in cellulose relative to other *Chlorocebus* species. Our results also support the notion that, in general, ecological conditions and phylogeny rather than geographical proximity cause differentiation of the GI microbiota, with no detectable correlation between the mean pairwise distances between the GI microbiotas of the various *Chlorocebus* populations and the physical distance between their habitats. Within the grivet and vervet populations our results indicate that ecology was more important than phylogeny, with samples generally structuring according to population rather than species (Supplementary Fig. 4). Notably, the GI microbiota profiles of the population at the southernmost sampling site, Arba Minch, did not form one consistent cluster, and this site is thought to be a hybrid zone^7^. Effects of hybridization on the GI microbiome have rarely been documented^37,38^, and in our case we cannot say for sure whether the lack of clustering was due to hybrids, or that we were sampling a mixed population.

In 16S rRNA gene amplicon-based microbiota studies chloroplast sequences are normally treated as contaminants and steps are routinely taken to eliminate them^39,40^. 16S rRNA gene sequences are highly conserved, and the chloroplast version of this gene is not commonly used as a marker in phylogenetic studies due to poor resolution on lower taxonomic levels, although it has been used for deep branching lineages^41^. Thus, results from this part of the study should be interpreted with some caution. Relating the relative abundances of specific OTUs with the relative abundance of sequence reads classified as Poaceae, in individual Bale monkeys, only identified 9 relatively rare OTUs (Supplementary Table 9) with a significant correlation. Most likely the number of chloroplast reads recovered from a sample from a specific individual only reflects feeding behaviour on a very short time scale. Within each of the three habitats under study here, Individual Bale monkeys have very similar diets, and the observed composition of the GI microbiota in a given sample may not be an accurate reflection of short term feeding behaviour. On the population level, however, the trends observed in our chloroplast data make good sense in terms of feeding behaviour both across sampling sites and seasons (Fig. 6 and Supplementary Fig. 7).

In this study we have conducted the first analysis of the microbiota of the Ethiopian Bale monkey, one of very few primate species of bamboo specialists. We have shown that anthropogenic degradation of their habitats has the potential to cause an ecologically plastic response in their GI microbiota on the local scale. Whether this response is beneficial to the host is uncertain. We have further shown that the Bale monkey GI microbiota can have a strong seasonal signal, but that this signal may be partially disrupted by human encroachment. We also provide evidence that in spite of moving towards a more generalist lifestyle, the Bale monkey GI microbiota retains a distinct species signature relative to other *Chlorocebus* monkeys. Finally, we found that chloroplast sequence reads, produced as a by-product of GI microbiota 16S rRNA gene amplicon sequencing, can potentially provide useful information about the diversity of green plants in animal diets.

## Methods

### Study sites

The three Bale monkey populations in this study live in either the continuous Odobullu Forest (CBF = continuous bamoboo forest) or one of the two fragmented forests Afursa (NBF = no bamboo forest) and Kokosa (DBF = degraded bamboo forest) in the southern Ethiopian Highlands (Fig. 2, Table 1). CBF is dominated by bamboo, but also has tree-dominated sections, shrubland and occasional stretches of grassland^42^. DBF and NBF lie ~160 km west of CBF. DBF consists of degraded bamboo forest with large trees set amidst a matrix of human settlements, cultivated land, shrubland and grazing land. NBF is set upon a hilltop and is a mix of secondary forest, shrubland and *Eucalyptus* plantation with grass cover underneath, and it is surrounded by an anthropogenic matrix including cultivated lands, pastures, and human settlements^15^. Bamboo here has been nearly extirpated. Both DBF and NBF were dominated by bamboo forest only three decades ago, and although they are only 9 km apart they have been separated by human settlements and agriculture for many decades^8^.

**Table 1:** Sample overview. SNNPR = Southern Nations, Nationalities, and Peoples’ Region.

Population	Region	Species	Habitat type	Human influence	#samples
Afursa (NBF)	SNNPR	Bale monkey	Forest fragment	Intermediate	66
Kokosa (DBF)	Oromia	Bale monkey	Forest fragment	High	63
Odobullu (CBF)	Oromia	Bale monkey	Continuous forest	Low	29
Asella	Oromia	Grivet	Plantation/forest fragment	High	12
Awassa	SNNPR	Grivet	Woodland	High	13
Wondo Genet	SNNPR	Grivet	Plantation/natural forest	High	14
Arba Minch	SNNPR	Vervet/Grivet	Plantation/natural forest	High	11
Sof Omar	Oromia	Vervet	Tree dominated forest	High	11

Samples from vervet and grivet monkeys were collected from 5 sites in the Oromia and Southern Nations, Nationalities, and Peoples’ (SNNP) regions surrounding the current range of the Bale monkeys (Fig. 2, Table 1). All of these five sites represent habitats with a high degree of human contact.

### Sampling and DNA extraction

Fecal samples from Bale monkeys were collected from October 2013 to June 2014 covering both the wet and dry seasons. In addition, we collected feces from vervet and grivet monkeys once at each of the five sites in 2016. We collected fresh feces immediately after defecation to avoid contamination. To minimize the risk of repeated fecal sampling from a single individual, we collected samples within a short period of time, for up to five consecutive days per month. For each sample approximately 2 grams of fecal material was transferred to a 15 ml sterile plastic vial with 8–10 ml 97% ethanol. Sample tubes were then stored at Addis Ababa University pending transport to the University of Oslo. DNA extraction from the fecal samples was carried out with the PowerSoil 96 well DNA isolation kit (MO BIO Laboratories Inc., Carlsbad, CA, USA). Permission to conduct this research was granted by the Ethiopian Wildlife Conservation Authority in compliance with the Convention on International Trade in Endangered Species of Wild Fauna and Flora (CITES). Fecal samples were collected non-invasively without harming or disturbing the animals. This study meets all animal care policies and adheres to the legal requirements of Ethiopia and Norway. It also complied with the ethical and legal requirements of the American Society of Primatologists Principles for the Ethical Treatment of Nonhuman Primates.

### Illumina sequencing

Library preparation for Illumina sequencing of the V4 region of the 16S rRNA gene was carried out according to de Muinck *et al*.^43^, using the primers 515 f (GTGYCAGCMGCCGCGGTAA) and 806r (GGACTACNVGGGTWTCTAAT). Sequencing was carried out on an Illumina HiSeq. 2500 apparatus (Illumina, San Diego, CA, USA) using the 2 × 250PE rapid run mode. An overview of the sequencing libraries can be found in Supplementary Table 10.

The total raw number of 16S rRNA gene sequence reads after quality trimming and paired read merging was 30,092,962, and the mean per sample read number was 137,411 (±36,180 s.d.). Importantly, 1,349,220 (4.5%) of the sequence reads were classified as eukaryotic chloroplast DNA. These reads were removed before analysis of the bacterial/archaeal communities and analyzed separately.

### Data processing and analyses

Low quality reads were trimmed and Illumina adapters were removed using Trimmomatic v0.36^44^ with default settings. Reads mapping to the PhiX genome (NCBI id: NC_001422.1) were removed using BBMap v36.02^45^. De-multiplexing of data based on the dual index sequences was carried out using custom scripts^43^. Internal barcodes and spacers were removed using cutadapt v1.4.1^46^ and paired reads were merged using FLASH v1.2.11^47^ with default settings.

Further processing of sequence data was carried out using a combination of vsearch v2.0.3^48^ and usearch v8.1.1861^49^. Specifically, dereplication was performed with the ‘derep_fulllength’ function in vsearch with the minimum unique group size set to 2. Operational taxonomic unit (OTU) clustering, chimera removal, taxonomic assignment and OTU table building were carried out using the uparse pipeline^50^. Taxonomic assignment to the genus level was done against the RDP-15 training set. Classification was generally poor, with only 9.3% classified to the genus level with a confidence of 0.9 or higher and 63.3% classified to the phylum level with that degree of confidence. Between-sample differences in sequencing library size were normalized by common scaling^51^. After library scaling the data were filtered to retain only OTUs with at least 0.1% relative abundance (corresponding to 48 reads in the common scaled data) in at least one sample. These filtering criteria are used throughout unless other criteria are specifically stated. The total number of 97% bacterial/archaeal OTUs, in the raw data was 4289. After filtering, in order to eliminate OTU artefacts, the number dropped to 1298.

1,349,220 (4.5%) of the 16S rRNA gene sequences were classified as eukaryote chloroplasts. These sequences were further classified to 141 OTUs. For analysis of reads classified as chloroplast sequence, OTUs with fewer than a total of 1000 reads were removed from the data, as well as samples where chloroplast reads made up less than 1% of the total, leaving 106 (n = 55 for NBF, n = 32 for DBF, and n = 19 for CBF) samples and 20 OTUs that made up more than 99% of total chloroplast sequences. Reads in each sample were normalized to the original library size. Further classification of sequences was done manually by BLAST search^52^ against the GenBank nucleotide collection. We assigned the chloroplast OTUs to taxonomic groups based on the top hits from different publication events. The taxonomic level of these groups relied on the degree of consistency between the hits for each OTU. Accessions for uncultured organism clones were ignored.

All statistical analyses were done in R^53^. Permutational Multivariate Analysis of Variance Using Distance Matrices (PERMANOVA) and Analysis of Similarities (ANOSIM) were carried out using the’adonis’ and ‘anosim’ functions in the ‘vegan’ package, respectively, with Bray-Curtis dissimilarities and 10,000 permutations. Non-metric multidimensional scaling (NMDS) of Bray Curtis distance matrices was carried out using the ‘isoMDS’ function in the ‘MASS’ package. We used Spearman correlations, as implemented in the function ‘cor.test’ to test for associations between chloroplast abundances and specific OTUs, applying the Bonferroni correction for multiple hypothesis testing. To test whether populations at sampling sites that are closer together have more similar GI microbiotas than populations at sites further apart we computed mean Bray-Curtis distances between populations in all possible pairwise combinations of the eight sites. The resulting vector of distances was then tested for significant Spearman correlation with the geographical distances (km) between the corresponding pairs of sites. Exact tests for differences in means between two groups of negative binomially distributed counts were carried out using the edgeR package^51,54^. For these tests we did not use common scaled data, as library size differences are accounted for as part of the statistical algorithm. Specific filtering criteria were used for exact tests in order to focus on abundant OTUs present in many individuals, i.e. an OTU had to be observed at least 0.1% in at least one third of the samples in the data set being analyzed.

### Data availability

All sequence data produced in this study are available at the NCBI Sequence Read Archive under BioProject ID PRJNA407723.

## Electronic supplementary material

Supplementary information
