# Supplementary material for: Ecological plasticity in the gastrointestinal microbiomes of Ethiopian Chlorocebus monkeys
Source: Sci Rep. 2018 Jan 8;8:20. doi: 10.1038/s41598-017-18435-2 (PMC5758796; doi:10.1038/s41598-017-18435-2)
Supplement: Supplementary file 1 — Supplementary information [file 41598_2017_18435_MOESM1_ESM.pdf]

Ecological plasticity in the gastrointestinal microbiomes of Ethiopian *Chlorocebus* monkeys

Pål Trosvik[1*], Eli K Rueness[1], Eric J de Muinck[1], Amera Moges[2,3] and Addisu Mekonnen[1,3]

[1]Centre for Ecological and Evolutionary Synthesis (CEES), Department of Biosciences, University of Oslo, Norway; [2]Department of Biology, Bahir Dar University, Bahir Dar, Ethiopia and [3]Department of Zoological Sciences, Addis Ababa University, Addis Ababa, Ethiopia

Supplementary information: Supplementary figures 1-8. Supplementary tables 1-10.

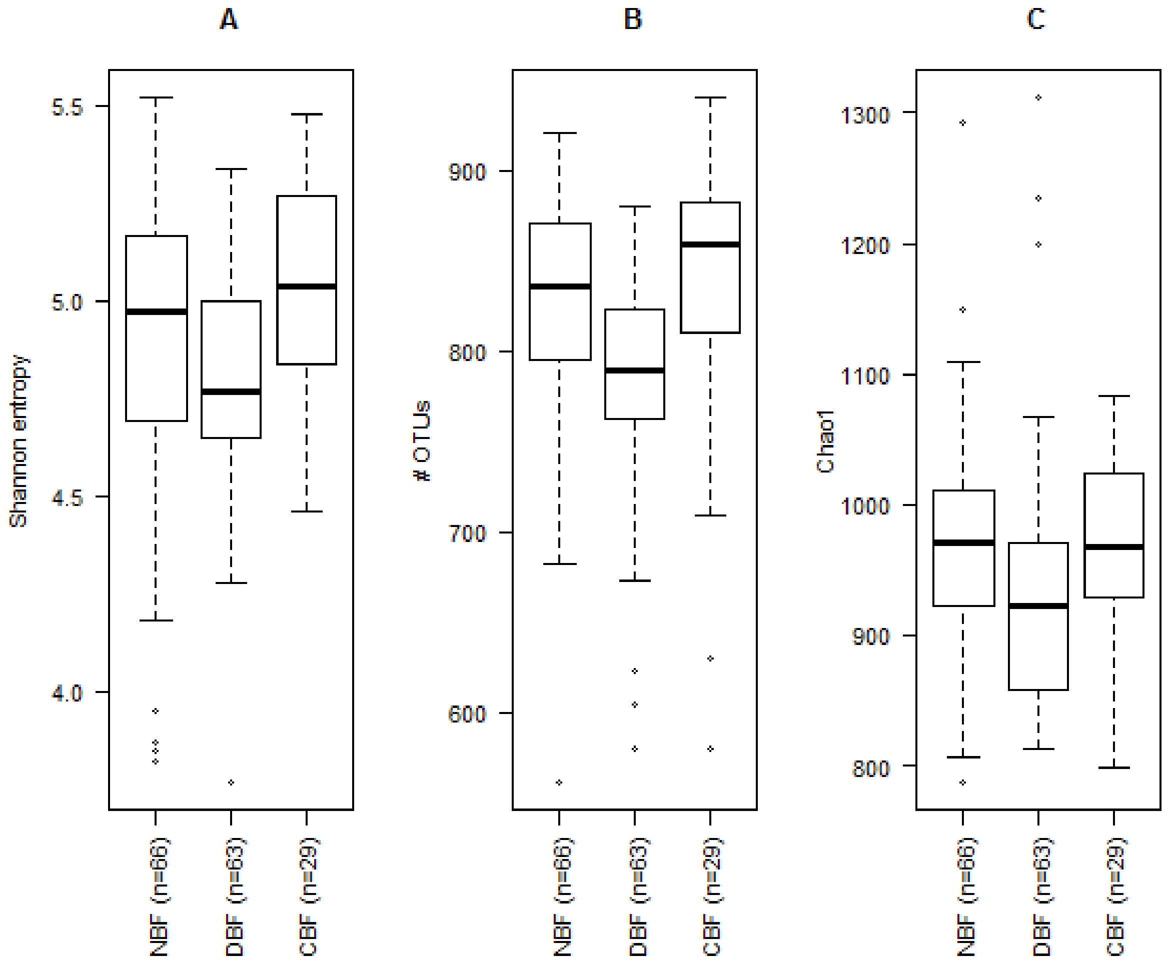

Supplementary Fig. 1. OTU diversity in the three Bale monkey populations. (A) Shannon entropy, (B) richness (C) Chao1 diversity estiamtor. Each box represents the interquartile range while the whiskers represent 1.5 times the interquartile range. Points outside the whiskers represent outliers. NBF=no bamboo forest, DBF=degraded bamboo forest, CBF=continuous bamboo forest.

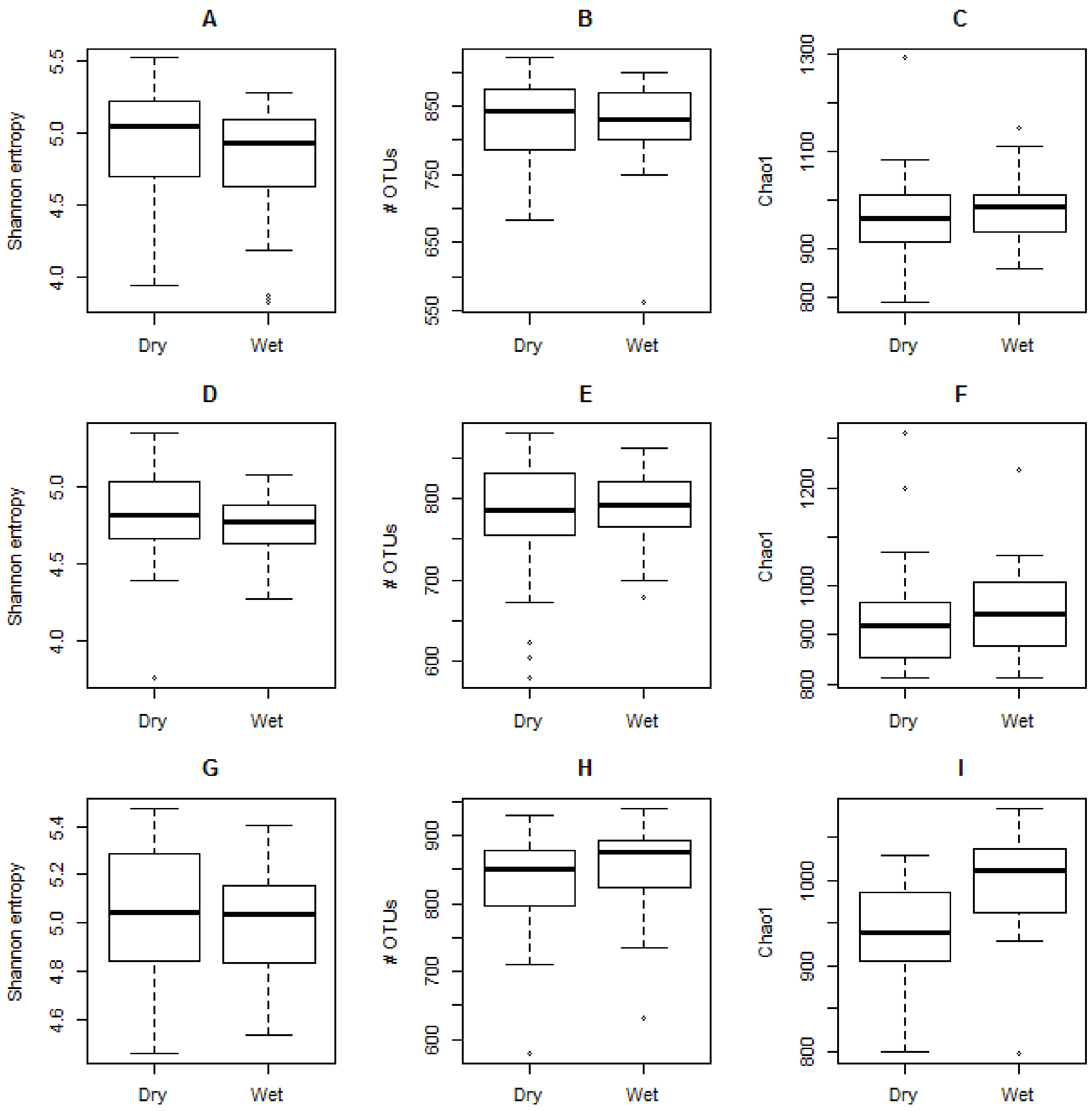

Supplementary Fig. 2. Divsersity of the GI microbiota in the Bale monkey populations at (A-C) NBF, (D-F) DBF, and (G-H) CBF. Each box represents the interquartile range while the whiskers represent 1.5 times the interquartile range. Points outside the whiskers represent outliers.

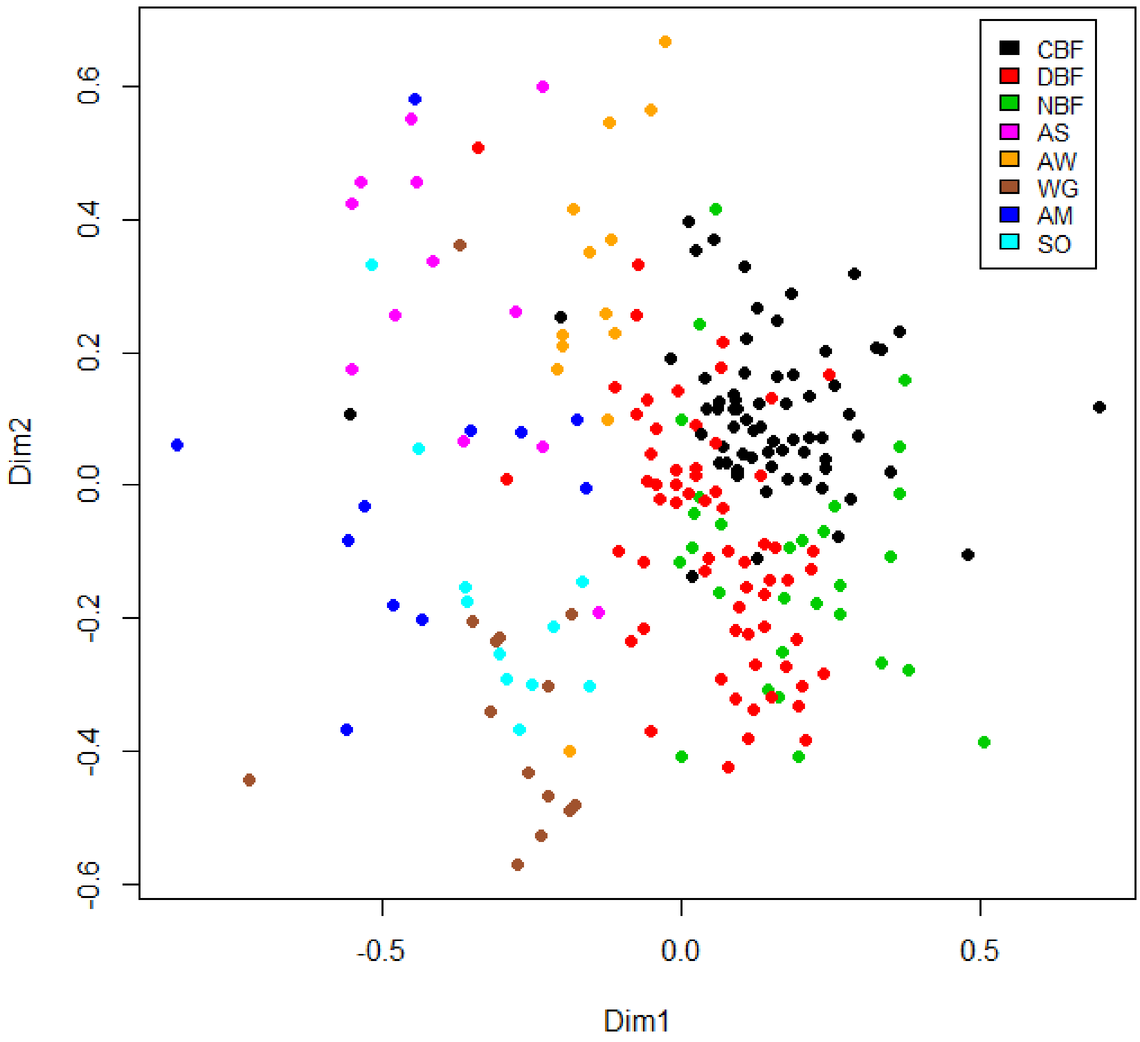

Supplementary Fig. 3. NMDS of all chlorocebus populations. NMDS of 158 Bale monkey samples from all three populations (NBF=no bamboo forest, DBF=degraded bamboo forest, CBF=continuous bamboo forest), 39 samples from three grivet populations (AS=Asella, AW=Awassa, WG=Wondo Genet), 11 samples from one vervet population (SO=Sof Omar) and 11 samples from a mixed population with hybrids (AM=Arba Minch). The plot shows the two main dimensions of variation, with dots colour coded according to sampling site (top right corner).

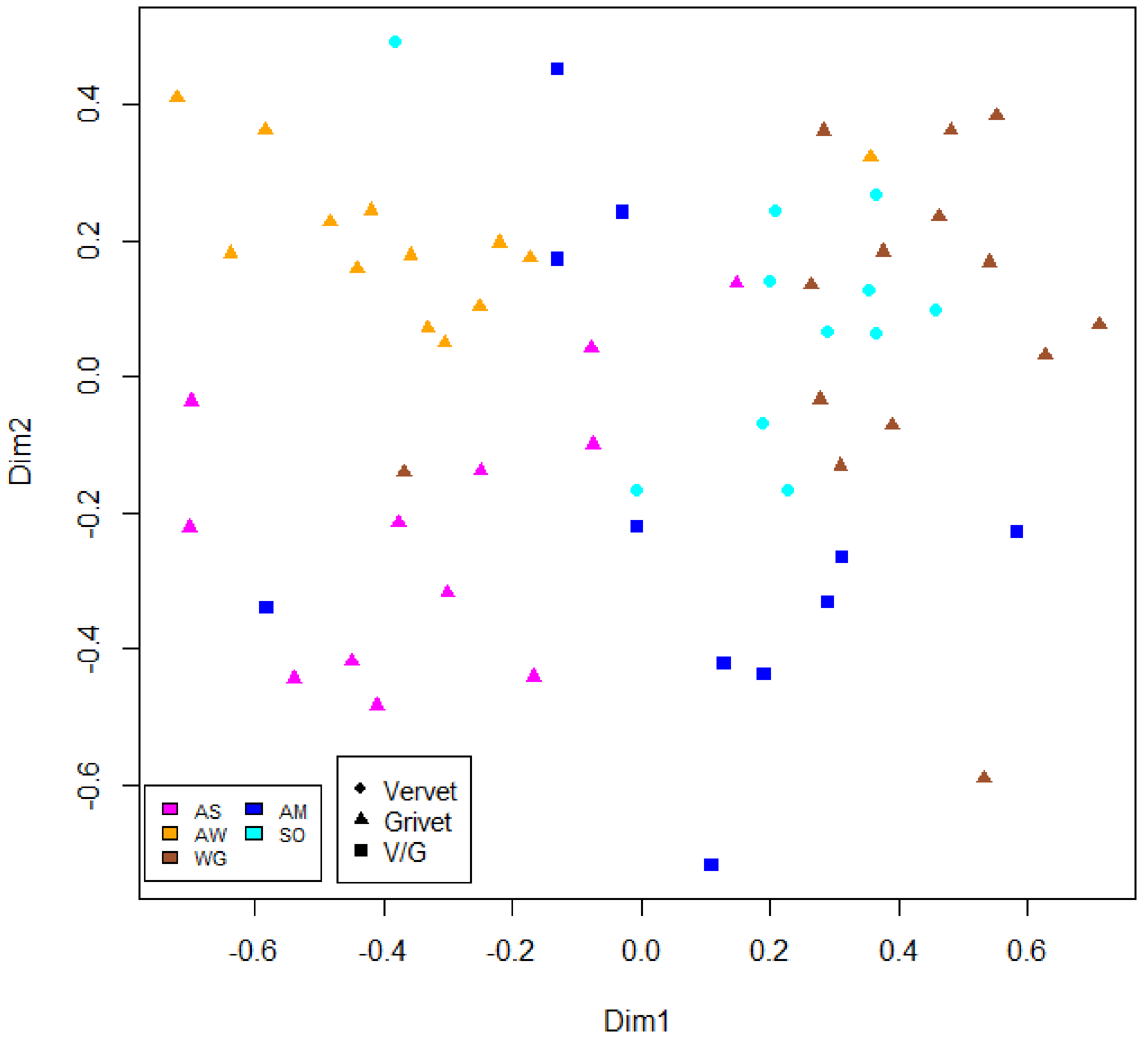

Supplementary Fig. 4. NMDS of grivet and vervet populations. NMDS of samples from three grivet populations (AS=Asella (n=12), AW=Awassa (n=13), WG=Wondo Genet (n=14)), one vervet population (SO=Sof Omar (n=11)) and one mixed population with hybrids (Arba Minch (n=11). The plot shows the two main dimensions of variation, with plotted characters colour coded according to sampling site and shaped according to species (bottom left corner).

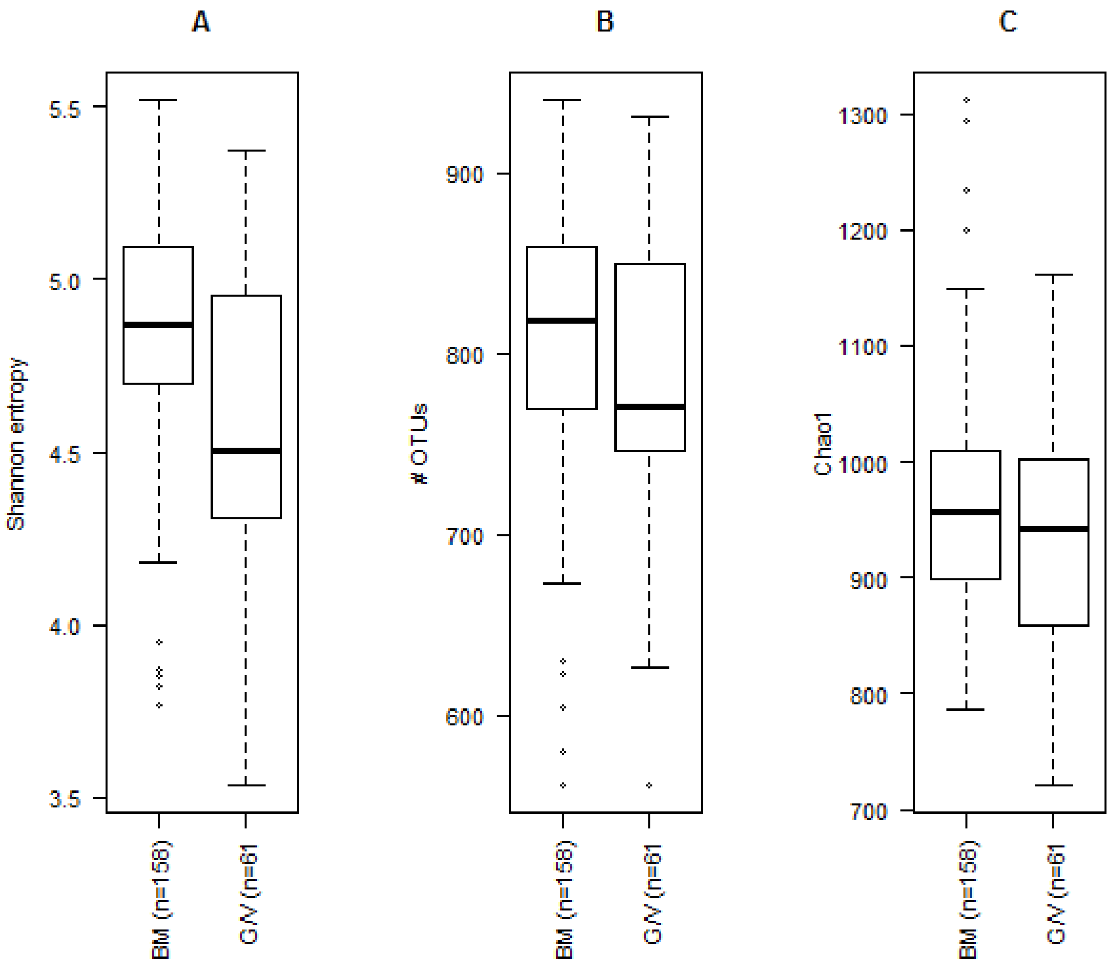

Supplementary Fig. 5. OTU diversity in all three Bale monkey combined and all five grivet and vervet populations combined. (A) Shannon entropy, (B) OUT richness, and (C) Chao1 diversity estimator. Each box represents the interquartile range while the whiskers represent 1.5 times the interquartile range. Points outside the whiskers represent outliers. BM=Bale monkey, G/V=vervet/grivet.

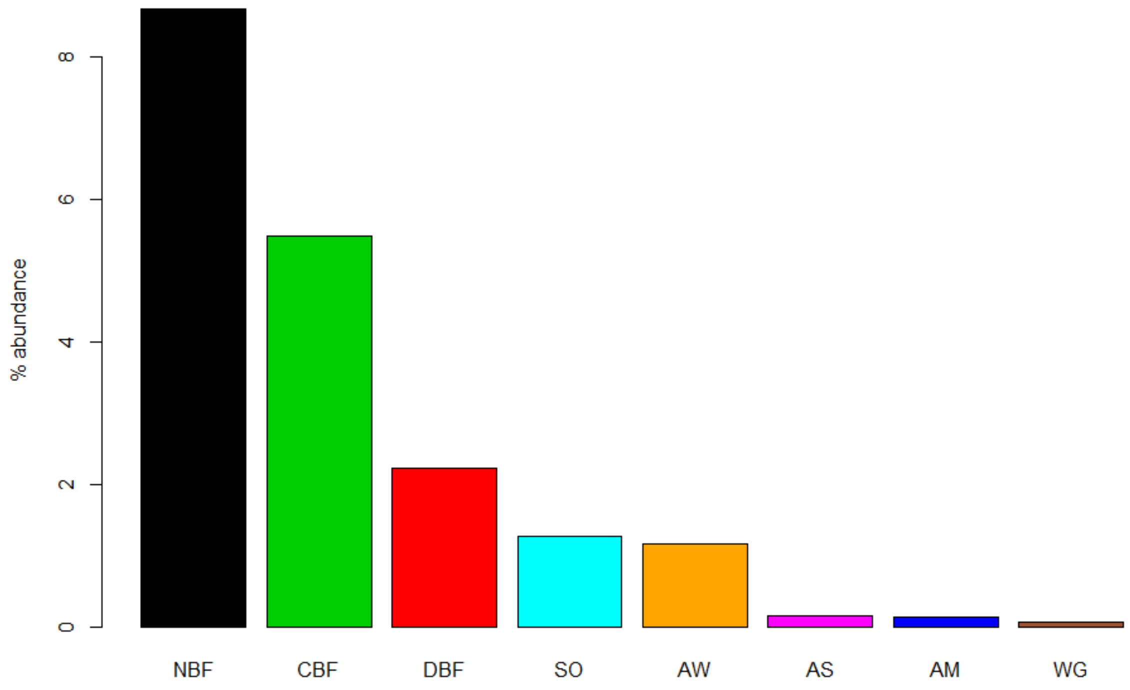

Supplementary Fig. 6. Percentage of total reads classified as chloroplasts at each sampling site. Sites are presented as highest (left) to lowest percentage (right). NBF=no bamboo forest,CBF=continuous bamboo forest, DBF=degraded bamboo forest, SO=Sof Omar, AW=Awassa, AS=Asella, AM=Arba Minch, WG=Wondo Genet.

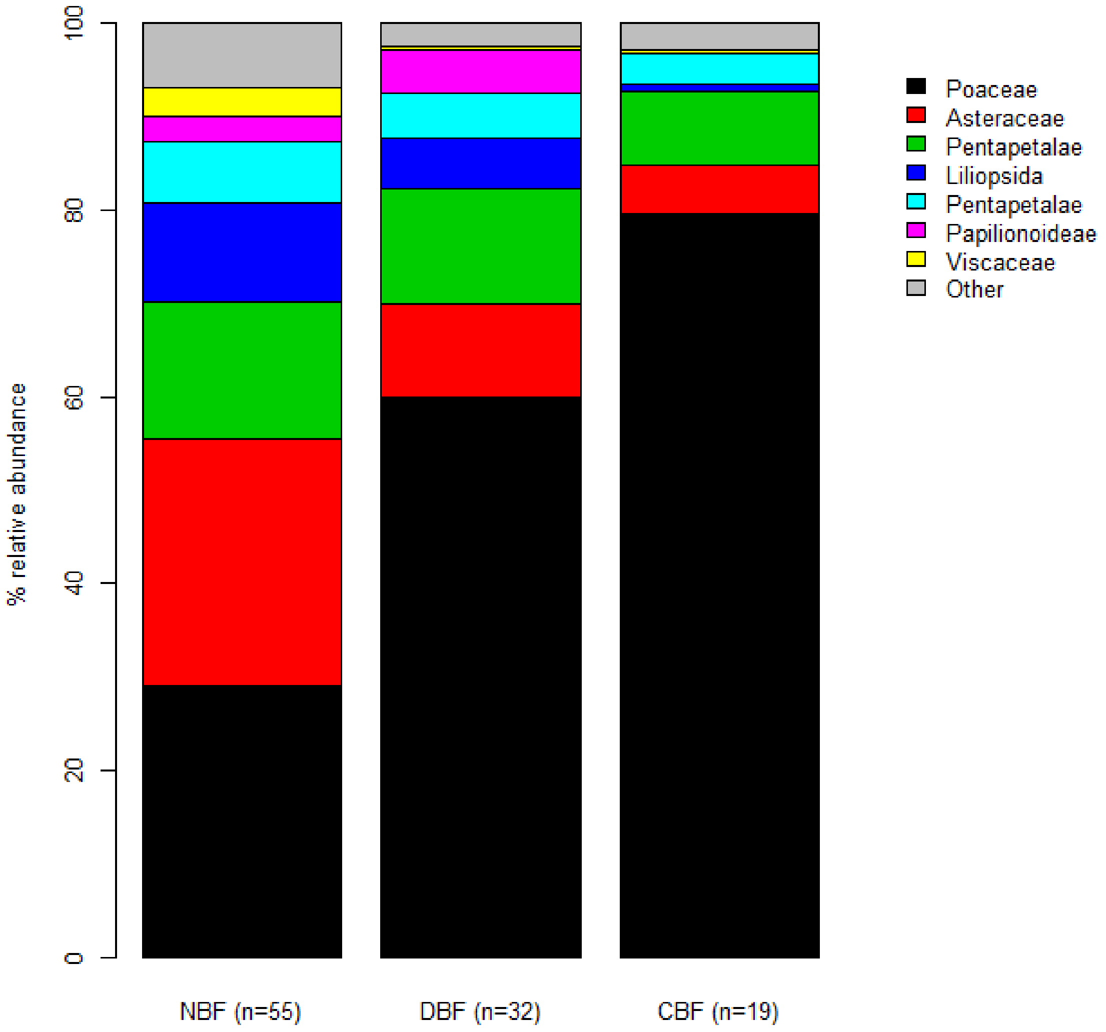

Supplementary Fig. 7. Relative abundances of the seven top OTUs classified as chloroplasts in the sequence data from the three Bale monkey populations. The colour key and classification shown on the right hand side give only the high level taxonomy (family and up). Poaceae (Gramineae) is the family of plants that includes grasses such as bamboo. Asteraceae (Compositae) is a very large family of flowering plants that includes dandelions and daisies. Pentapetalae is a high level clade that includes e.g. the families Asteraceae and Rosaceae. Liliopsida is a class containing the lily family. Papilionoideae (Faboideae) is a subfamily of Fabaceae which includes legumes. Viscaceae (Santalaceae) is a family of flowering plants containing mistletoes and sandalwood. NBF = no bamboo forest, DBF = degraded bamboo forest, CBF = continuous bamboo forest.

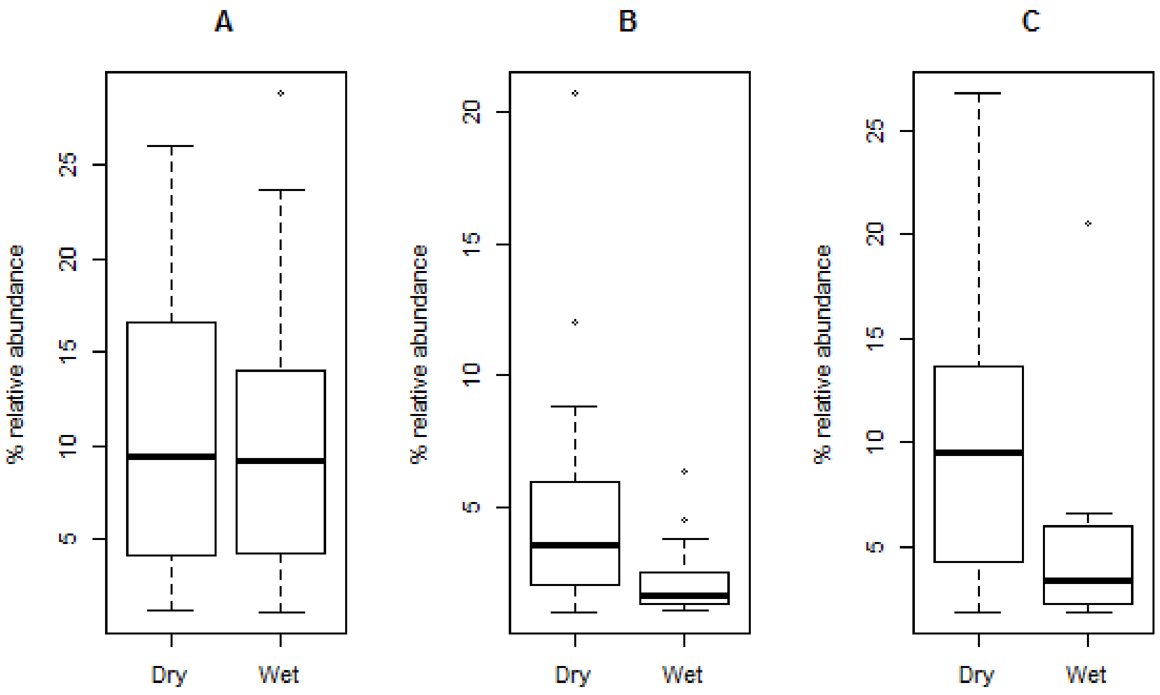

Supplementary Fig. 8. Percentage of total reads classified as chloroplasts from each of the three Bale monkey populations split into samples collected during the dry and wet seasons. (A) NBF (p=0.66, Wilcoxon rank sum test), (B) DBF (p=0.04), (C) CBF (p=0.13). The lines in the boxes are medians. Each box represents the interquartile range while the whiskers represent 1.5 times the interquartile range. Points outside the whiskers represent outliers.

Table S1. Results from exact tests showing OTUs with significantly different occurrence in Kokosa relative to Afursa, including full taxonomic assignment, log-fold difference in abundance (logFC), test p-value and false discovery rate (FDR).

OTU#	Taxonomy	logFC	PValue	FDR
OTU56	"d:Bacteria(1.0000),p:Bacteroidetes(0.9930),c:Bacteroidia(0.8348),o:Bacteroidales(0.4658),f:Porphyromonadaceae(0.3068),g:Parabacteroides(0.0215);"	8.414475	1.34E-80	2.26E-78
OTU26	"d:Bacteria(1.0000),p:Tenericutes(0.4731),c:Mollicutes(0.2113),o:Anaeroplasmatales(0.0945),f:Anaeroplasmataceae(0.0296),g:Asteroleplasma(0.0094);"	8.69081	2.21E-60	1.86E-58
OTU185	"d:Bacteria(1.0000),p:Firmicutes(0.8620),c:Clostridia(0.5845),o:Clostridiales(0.3031),f:Gracilibacteraceae(0.0883),g:Gracilibacter(0.0167);"	2.321396	1.70E-17	9.51E-16
OTU117	"d:Bacteria(1.0000),p:Firmicutes(0.7674),c:Clostridia(0.3967),o:Clostridiales(0.1942),f:Lachnospiraceae(0.0454),g:Coprococcus(0.0119);"	2.845983	4.87E-16	2.05E-14
OTU22	"d:Bacteria(1.0000),p:Bacteroidetes(0.9922),c:Bacteroidia(0.8348),o:Bacteroidales(0.4658),f:Prevotellaceae(0.1525),g:Paraprevotella(0.0295);"	2.249803	1.05E-14	3.52E-13
OTU65	"d:Bacteria(1.0000),p:Firmicutes(0.9914),c:Clostridia(0.8746),o:Clostridiales(0.6422),f:Ruminococcaceae(0.3068),g:Papillibacter(0.0295);"	2.34319	1.61E-14	4.52E-13
OTU90	"d:Bacteria(1.0000),p:Firmicutes(0.9970),c:Clostridia(0.9207),o:Clostridiales(0.8197),f:Eubacteriaceae(0.4275),g:Eubacterium(0.1672);"	2.144572	3.59E-14	8.61E-13
OTU17	"d:Bacteria(1.0000),p:Tenericutes(0.8384),c:Mollicutes(0.5845),o:Acholeplasmatales(0.2305),f:Acholeplasmataceae(0.1097),g:Acholeplasma(0.0191);"	-2.55199	4.47E-14	9.38E-13
OTU64	"d:Bacteria(1.0000),p:Firmicutes(0.7911),c:Erysipelotrichia(0.3967),o:Erysipelotrichales(0.1942),f:Erysipelotrichaceae(0.0883),g:Clostridium_XVIII(0.0167);"	2.69489	1.17E-13	2.18E-12
OTU73	"d:Bacteria(1.0000),p:Proteobacteria(0.7674),c:Alphaproteobacteria(0.2113),o:Kiloniellales(0.0400),f:Kiloniellaceae(0.0140),g:Kiloniella(0.0060);"	-3.53759	1.63E-13	2.73E-12
OTU83	"d:Bacteria(1.0000),p:Bacteroidetes(0.8857),c:Flavobacteriia(0.2113),o:Flavobacteriales(0.0945),f:Flavobacteriaceae(0.0296),g:Riemerella(0.0094);"	3.069964	4.95E-12	7.55E-11
OTU122	"d:Bacteria(1.0000),p:Firmicutes(0.9999),c:Bacilli(0.9668),o:Lactobacillales(0.9457),f:Lactobacillaceae(0.9026),g:Lactobacillus(0.8158);"	-2.05842	1.20E-11	1.67E-10
OTU31	"d:Bacteria(1.0000),p:Bacteroidetes(0.9987),c:Bacteroidia(0.9704),o:Bacteroidales(0.9597),f:Rikenellaceae(0.9109),g:Alistipes(0.7814);"	1.868154	3.84E-11	4.96E-10
OTU87	"d:Bacteria(1.0000),p:Bacteroidetes(0.9922),c:Bacteroidia(0.8348),o:Bacteroidales(0.4658),f:Porphyromonadaceae(0.3068),g:Barnesiella(0.0215);"	1.832163	1.53E-10	1.83E-09
OTU45	"d:Bacteria(0.6389),p:Lentisphaerae(0.0530),c:Lentisphaeria(0.0165),o:Victivallales(0.0062),f:Victivallaceae(0.0015),g:Victivallis(0.0017);"	-2.27918	2.51E-10	2.81E-09
OTU85	"d:Bacteria(1.0000),p:Bacteroidetes(0.8384),c:Bacteroidia(0.3342),o:Bacteroidales(0.1579),f:Bacteroidaceae(0.0042),g:Bacteroides(0.0034);"	2.953541	2.73E-10	2.82E-09
OTU135	"d:Bacteria(1.0000),p:Tenericutes(0.4731),c:Mollicutes(0.2113),o:Acholeplasmatales(0.0945),f:Acholeplasmataceae(0.0296),g:Acholeplasma(0.0094);"	2.915778	2.86E-10	2.82E-09
OTU33	"d:Bacteria(1.0000),p:Bacteroidetes(0.7911),c:Bacteroidia(0.0345),o:Bacteroidales(0.0136),f:Porphyromonadaceae(0.0061),g:Barnesiella(0.0043);"	1.88703	3.71E-10	3.46E-09
OTU219	"d:Bacteria(1.0000),p:Firmicutes(0.9802),c:Clostridia(0.8348),o:Clostridiales(0.4658),f:Lachnospiraceae(0.1525),g:Lachnospiracea_incertae_sedis(0.0295);"	-1.85229	5.75E-10	5.08E-09
OTU520	"d:Bacteria(1.0000),p:Firmicutes(0.7911),c:Clostridia(0.4593),o:Clostridiales(0.2305),f:Clostridiaceae_1(0.0668),g:Clostridium_sensu_stricto(0.0143);"	-1.54175	9.81E-10	8.24E-09
OTU92	"d:Bacteria(1.0000),p:Firmicutes(0.9993),c:Clostridia(0.9391),o:Clostridiales(0.8477),f:Ruminococcaceae(0.6553),g:Ruminococcus(0.1672);"	-2.13053	1.79E-09	1.40E-08
OTU181	"d:Bacteria(1.0000),p:Firmicutes(0.9983),c:Clostridia(0.9929),o:Clostridiales(0.9841),f:Lachnospiraceae(0.9274),g:Pseudobutyrivibrio(0.7126);"	-1.70877	1.83E-09	1.40E-08
OTU217	"d:Bacteria(1.0000),p:Firmicutes(0.3677),c:Clostridia(0.1500),o:Clostridiales(0.0673),f:Ruminococcaceae(0.0140),g:Ruminococcus(0.0060);"	2.531425	2.68E-09	1.96E-08
OTU20	"d:Bacteria(1.0000),p:Tenericutes(0.9954),c:Mollicutes(0.9299),o:Anaeroplasmatales(0.7305),f:Anaeroplasmataceae(0.5430),g:Anaeroplasma(0.3238);"	-2.52331	3.02E-09	2.11E-08
OTU164	"d:Bacteria(1.0000),p:Firmicutes(0.9954),c:Clostridia(0.7097),o:Clostridiales(0.3775),f:Lachnospiraceae(0.1861),g:Syntrophococcus(0.0409);"	-1.66139	1.14E-08	7.68E-08
OTU50	"d:Bacteria(1.0000),p:Actinobacteria(1.0000),c:Actinobacteria(1.0000),o:Bifidobacteriales(0.9966),f:Bifidobacteriaceae(0.9970),g:Bifidobacterium(0.9871);"	2.541903	1.44E-08	9.27E-08
OTU19	"d:Bacteria(1.0000),p:Bacteroidetes(0.9988),c:Bacteroidia(0.8654),o:Bacteroidales(0.5981),f:Porphyromonadaceae(0.4275),g:Parabacteroides(0.0409);"	1.804257	1.72E-08	1.07E-07
OTU835	"d:Bacteria(1.0000),p:Bacteroidetes(0.9914),c:Bacteroidia(0.5845),o:Bacteroidales(0.3031),f:Porphyromonadaceae(0.1311),g:Paludibacter(0.0215);"	2.374534	6.33E-08	3.80E-07
OTU228	"d:Bacteria(1.0000),p:Firmicutes(0.9987),c:Clostridia(0.9522),o:Clostridiales(0.8897),f:Lachnospiraceae(0.7302),g:Anaerostipes(0.5751);"	-1.87894	1.62E-07	9.40E-07
OTU8	"d:Bacteria(1.0000),p:Bacteroidetes(0.9899),c:Sphingobacteriia(0.3342),o:Sphingobacteriales(0.1579),f:Sphingobacteriaceae(0.0668),g:Mucilaginibacter(0.0143);"	1.92737	2.13E-07	1.19E-06
OTU312	"d:Bacteria(1.0000),p:Firmicutes(0.8620),c:Clostridia(0.6471),o:Clostridiales(0.3394),f:Clostridiaceae_2(0.0883),g:Alkaliphilus(0.0167);"	-1.17208	2.52E-07	1.36E-06
OTU3	"d:Bacteria(1.0000),p:Bacteroidetes(0.9914),c:Bacteroidia(0.6471),o:Bacteroidales(0.3394),f:Porphyromonadaceae(0.1525),g:Paludibacter(0.0215);"	1.576312	3.85E-07	2.02E-06
OTU374	"d:Bacteria(1.0000),p:Bacteroidetes(0.9999),c:Bacteroidia(0.9988),o:Bacteroidales(0.9993),f:Prevotellaceae(0.9898),g:Prevotella(0.9914);"	1.503511	4.60E-07	2.34E-06
OTU208	"d:Bacteria(1.0000),p:Firmicutes(0.8384),c:Clostridia(0.5845),o:Clostridiales(0.1942),f:Ruminococcaceae(0.0668),g:Clostridium_III(0.0119);"	-1.48735	8.35E-07	4.12E-06
OTU2	"d:Bacteria(1.0000),p:Firmicutes(0.9987),c:Clostridia(0.9943),o:Clostridiales(0.9954),f:Clostridiaceae_1(0.8778),g:Clostridium_sensu_stricto(0.7126);"	-2.08635	9.71E-07	4.66E-06
OTU141	"d:Bacteria(0.9379),p:Firmicutes(0.1875),c:Clostridia(0.0315),o:Clostridiales(0.0123),f:Clostridiaceae_1(0.0037),g:Clostridium_sensu_stricto(0.0031);"	-2.58215	1.01E-06	4.71E-06
OTU98	"d:Bacteria(1.0000),p:Firmicutes(0.7674),c:Clostridia(0.2725),o:Clostridiales(0.1218),f:Lachnospiraceae(0.0454),g:Anaerostipes(0.0119);"	1.252232	1.43E-06	6.51E-06
OTU34	"d:Bacteria(1.0000),p:Proteobacteria(1.0000),c:Epsilonproteobacteria(1.0000),o:Campylobacterales(1.0000),f:Helicobacteraceae(0.9973),g:Helicobacter(0.9968);"	1.340366	2.67E-06	1.15E-05
OTU147	"d:Bacteria(1.0000),p:Firmicutes(0.9988),c:Clostridia(0.9559),o:Clostridiales(0.9037),f:Lachnospiraceae(0.7302),g:Clostridium_XlVa(0.4804);"	-1.50773	2.72E-06	1.15E-05
OTU21	"d:Bacteria(1.0000),p:Proteobacteria(0.9987),c:Betaproteobacteria(0.9115),o:Rhodocyclales(0.5981),f:Rhodocyclaceae(0.4275),g:Denitratisoma(0.1672);"	-1.1549	2.75E-06	1.15E-05
OTU106	"d:Bacteria(1.0000),p:Firmicutes(0.8620),c:Clostridia(0.6471),o:Clostridiales(0.3394),f:Ruminococcaceae(0.1097),g:Ruminococcus(0.0143);"	1.428504	4.33E-06	1.77E-05
OTU5	"d:Bacteria(1.0000),p:Bacteroidetes(0.9988),c:Bacteroidia(0.9886),o:Bacteroidales(0.9757),f:Prevotellaceae(0.5055),g:Prevotella(0.1672);"	1.604439	4.54E-06	1.81E-05
OTU136	"d:Bacteria(1.0000),p:Tenericutes(0.2622),c:Mollicutes(0.0888),o:Entomoplasmatales(0.0400),f:Spiroplasmataceae(0.0140),g:Spiroplasma(0.0060);"	-1.45794	4.63E-06	1.81E-05
OTU235	"d:Bacteria(1.0000),p:Actinobacteria(0.9914),c:Actinobacteria(0.8562),o:Coriobacteriales(0.5540),f:Coriobacteriaceae(0.3872),g:Slackia(0.0295);"	-1.09751	8.44E-06	3.22E-05
OTU1734	"d:Bacteria(1.0000),p:Proteobacteria(0.3677),c:Deltaproteobacteria(0.1500),o:Bdellovibrionales(0.0673),f:Bdellovibrionaceae(0.0218),g:Vampirovibrio(0.0077);"	-2.06312	1.17E-05	4.33E-05
OTU143	"d:Bacteria(1.0000),p:Firmicutes(0.9994),c:Clostridia(0.9704),o:Clostridiales(0.9597),f:Lachnospiraceae(0.8051),g:Clostridium_XlVa(0.6095);"	-1.03216	1.19E-05	4.33E-05
OTU35	"d:Bacteria(1.0000),p:Firmicutes(0.8620),c:Clostridia(0.6471),o:Clostridiales(0.2668),f:Ruminococcaceae(0.1311),g:Intestinimonas(0.0191);"	2.045919	1.32E-05	4.67E-05
OTU93	"d:Bacteria(1.0000),p:Firmicutes(0.9978),c:Clostridia(0.9299),o:Clostridiales(0.8337),f:Ruminococcaceae(0.5430),g:Sporobacter(0.0865);"	0.9492	1.33E-05	4.67E-05

OTU159	"d:Bacteria(0.9753),p:Bacteroidetes(0.6840),c:Sphingobacteriia(0.0285),o:Sphingobacteriales(0.0111),f:Sphingobacteriaceae(0.0037),g:Parapedobacter(0.0031);"	1.965507	1.80E-05	6.19E-05
OTU30	"d:Bacteria(1.0000),p:Firmicutes(0.9992),c:Erysipelotrichia(0.9595),o:Erysipelotrichales(0.9177),f:Erysipelotrichaceae(0.8861),g:Clostridium_XVIII(0.3760);"	1.240984	1.97E-05	6.61E-05
OTU129	"d:Bacteria(1.0000),p:Firmicutes(0.7674),c:Clostridia(0.3967),o:Clostridiales(0.1942),f:Clostridiaceae_3(0.0668),g:Brassicibacter(0.0143);"	-1.4521	2.74E-05	9.04E-05
OTU61	"d:Bacteria(1.0000),p:Proteobacteria(0.4731),c:Deltaproteobacteria(0.2113),o:Bdellovibrionales(0.0945),f:Bdellovibrionaceae(0.0296),g:Vampirovibrio(0.0094);"	-1.41421	5.61E-05	0.000181
OTU133	"d:Bacteria(1.0000),p:Firmicutes(0.6840),c:Clostridia(0.3342),o:Clostridiales(0.1218),f:Ruminococcaceae(0.0296),g:Intestinimonas(0.0077);"	1.357058	7.85E-05	0.000249
OTU36	"d:Bacteria(1.0000),p:Bacteroidetes(0.9995),c:Bacteroidia(0.9973),o:Bacteroidales(0.9985),f:Prevotellaceae(0.9687),g:Prevotella(0.9722);"	1.020711	8.43E-05	0.000262
OTU49	"d:Bacteria(1.0000),p:Firmicutes(0.9978),c:Clostridia(0.9391),o:Clostridiales(0.8477),f:Ruminococcaceae(0.6179),g:Oscillibacter(0.0751);"	0.78532	9.46E-05	0.000289
OTU126	"d:Bacteria(1.0000),p:Firmicutes(0.7911),c:Clostridia(0.4593),o:Clostridiales(0.2305),f:Clostridiaceae_4(0.0296),g:Thermotalea(0.0094);"	1.074896	0.000136	0.000407
OTU104	"d:Bacteria(1.0000),p:Firmicutes(0.5785),c:Clostridia(0.2725),o:Clostridiales(0.1218),f:Ruminococcaceae(0.0218),g:Intestinimonas(0.0060);"	-1.50162	0.000154	0.000455
OTU115	"d:Bacteria(0.9753),p:Firmicutes(0.1875),c:Clostridia(0.0345),o:Clostridiales(0.0136),f:Clostridiaceae_1(0.0042),g:Caloramator(0.0034);"	1.368196	0.000178	0.000517
OTU25	"d:Bacteria(1.0000),p:Proteobacteria(0.3677),c:Deltaproteobacteria(0.1500),o:Bdellovibrionales(0.0673),f:Bdellovibrionaceae(0.0218),g:Vampirovibrio(0.0077);"	1.306584	0.000198	0.000563
OTU2966	"d:Bacteria(1.0000),p:Firmicutes(0.9899),c:Clostridia(0.5845),o:Clostridiales(0.3031),f:Ruminococcaceae(0.1525),g:Pseudobacteroides(0.0215);"	-0.70987	0.000203	0.000567
OTU42	"d:Bacteria(0.7884),p:Proteobacteria(0.1203),c:Deltaproteobacteria(0.0255),o:Bdellovibrionales(0.0099),f:Bdellovibrionaceae(0.0031),g:Vampirovibrio(0.0027);"	1.630783	0.000226	0.000622
OTU265	"d:Bacteria(1.0000),p:Firmicutes(0.9990),c:Clostridia(0.9559),o:Clostridiales(0.9037),f:Ruminococcaceae(0.8425),g:Clostridium_IV(0.6438);"	-1.44968	0.000244	0.000661
OTU132	"d:Bacteria(1.0000),p:Firmicutes(0.8620),c:Clostridia(0.6471),o:Clostridiales(0.3394),f:Clostridiaceae_2(0.0668),g:Alkaliphilus(0.0143);"	-1.34504	0.000287	0.00076
OTU177	"d:Bacteria(1.0000),p:Firmicutes(0.9986),c:Clostridia(0.9931),o:Clostridiales(0.9841),f:Ruminococcaceae(0.9687),g:Gemmiger(0.4282);"	-1.10236	0.00029	0.00076
OTU145	"d:Bacteria(1.0000),p:Firmicutes(0.5785),c:Erysipelotrichia(0.2113),o:Erysipelotrichales(0.0945),f:Erysipelotrichaceae(0.0296),g:Erysipelothrix(0.0094);"	1.471001	0.000452	0.001168
OTU62	"d:Bacteria(1.0000),p:Bacteroidetes(0.9329),c:Bacteroidia(0.8348),o:Bacteroidales(0.4658),f:Prevotellaceae(0.1097),g:Alloprevotella(0.0143);"	1.177416	0.000487	0.00124
OTU13	"d:Archaea(1.0000),p:Euryarchaeota(0.9938),c:Thermoplasmata(0.8654),o:Methanomassiliicoccales(0.4658),f:Methanomassiliicoccaceae(0.3068),g:Methanomassiliicoccus(0.0751);"	1.141677	0.000546	0.001368
OTU67	"d:Bacteria(1.0000),p:Firmicutes(0.6840),c:Clostridia(0.3342),o:Clostridiales(0.1579),f:Lachnospiraceae(0.0296),g:Coprococcus(0.0077);"	0.907359	0.000735	0.001815
OTU420	"d:Bacteria(1.0000),p:Firmicutes(0.9986),c:Clostridia(0.9115),o:Clostridiales(0.8057),f:Lachnospiraceae(0.5055),g:Clostridium_XlVa(0.2716);"	-0.85549	0.000759	0.001849
OTU2828	"d:Bacteria(1.0000),p:Firmicutes(0.9984),c:Clostridia(0.9937),o:Clostridiales(0.9926),f:Ruminococcaceae(0.9847),g:Faecalibacterium(0.8158);"	-0.82333	0.001001	0.002402
OTU112	"d:Bacteria(1.0000),p:Firmicutes(0.7911),c:Clostridia(0.4593),o:Clostridiales(0.2305),f:Lachnospiraceae(0.0668),g:Coprococcus(0.0143);"	0.870383	0.001018	0.00241
OTU1	"d:Bacteria(1.0000),p:Firmicutes(1.0000),c:Clostridia(0.9958),o:Clostridiales(0.9968),f:Clostridiaceae_1(0.9857),g:Sarcina(0.9722);"	-1.10501	0.001187	0.00277
OTU733	"d:Bacteria(1.0000),p:Firmicutes(0.9986),c:Clostridia(0.9886),o:Clostridiales(0.9757),f:Ruminococcaceae(0.9191),g:Ruminococcus(0.7814);"	-1.03631	0.001332	0.003065
OTU322	"d:Bacteria(1.0000),p:Firmicutes(0.9990),c:Clostridia(0.9956),o:Clostridiales(0.9966),f:Eubacteriaceae(0.9888),g:Eubacterium(0.9914);"	-0.90925	0.001518	0.003446
OTU179	"d:Bacteria(1.0000),p:Firmicutes(0.9962),c:Clostridia(0.9023),o:Clostridiales(0.7746),f:Ruminococcaceae(0.5055),g:Sporobacter(0.0751);"	0.704973	0.001572	0.003522
OTU188	"d:Bacteria(1.0000),p:Firmicutes(0.9914),c:Clostridia(0.8746),o:Clostridiales(0.5540),f:Ruminococcaceae(0.2665),g:Intestinimonas(0.0295);"	0.827054	0.00164	0.003626
OTU1160	"d:Bacteria(1.0000),p:Firmicutes(0.8384),c:Clostridia(0.5845),o:Clostridiales(0.3031),f:Ruminococcaceae(0.1311),g:Ruminococcus(0.0143);"	0.999041	0.00181	0.003949
OTU120	"d:Bacteria(0.6015),p:Firmicutes(0.1427),c:Erysipelotrichia(0.0285),o:Erysipelotrichales(0.0111),f:Erysipelotrichaceae(0.0037),g:Holdemania(0.0031);"	-0.92776	0.002181	0.00464
OTU43	"d:Bacteria(1.0000),p:Firmicutes(0.8147),c:Clostridia(0.5219),o:Clostridiales(0.2668),f:Ruminococcaceae(0.0668),g:Ruminococcus(0.0094);"	-1.10493	0.002182	0.00464
OTU39	"d:Bacteria(1.0000),p:Firmicutes(0.7674),c:Negativicutes(0.2725),o:Selenomonadales(0.1218),f:Veillonellaceae(0.0454),g:Selenomonas(0.0119);"	0.85714	0.002217	0.004657
OTU192	"d:Bacteria(1.0000),p:Firmicutes(0.9093),c:Bacilli(0.5219),o:Bacillales(0.2668),f:Paenibacillaceae_1(0.1097),g:Cohnella(0.0191);"	-0.62802	0.002612	0.005418
OTU485	"d:Bacteria(1.0000),p:Firmicutes(0.9991),c:Clostridia(0.9559),o:Clostridiales(0.9037),f:Lachnospiraceae(0.6928),g:Clostridium_XlVa(0.3760);"	-0.49563	0.004107	0.008414
OTU201	"d:Bacteria(1.0000),p:Firmicutes(0.8384),c:Clostridia(0.5845),o:Clostridiales(0.3031),f:Ruminococcaceae(0.1311),g:Ruminococcus(0.0191);"	-0.7377	0.004538	0.009186

Table S2. Results from exact tests showing OTUs with significantly different occurrence in Odobullu relative to Afursa, including full taxonomic assignment, log-fold difference in abundance (logFC), test p-value and false discovery rate (FDR).

OTU#	Taxonomy	logFC	PValue	FDR
OTU56	"d:Bacteria(1.0000),p:Bacteroidetes(0.9930),c:Bacteroidia(0.8348),o:Bacteroidales(0.4658),f:Porphyromonadaceae(0.3068),g:Parabacteroides(0.0215);"	7.992833	5.50E-70	9.25E-68
OTU26	"d:Bacteria(1.0000),p:Tenericutes(0.4731),c:Mollicutes(0.2113),o:Anaeroplasmatales(0.0945),f:Anaeroplasmataceae(0.0296),g:Asteroleplasma(0.0094);"	6.839458	2.30E-40	1.93E-38
OTU21	"d:Bacteria(1.0000),p:Proteobacteria(0.9987),c:Betaproteobacteria(0.9115),o:Rhodocyclales(0.5981),f:Rhodocyclaceae(0.4275),g:Denitratisoma(0.1672);"	-5.09581	1.26E-34	7.05E-33
OTU179	"d:Bacteria(1.0000),p:Firmicutes(0.9962),c:Clostridia(0.9023),o:Clostridiales(0.7746),f:Ruminococcaceae(0.5055),g:Sporobacter(0.0751);"	2.892471	1.57E-27	6.60E-26
OTU85	"d:Bacteria(1.0000),p:Bacteroidetes(0.8384),c:Bacteroidia(0.3342),o:Bacteroidales(0.1579),f:Bacteroidaceae(0.0042),g:Bacteroides(0.0034);"	5.237548	2.43E-21	8.16E-20
OTU835	"d:Bacteria(1.0000),p:Bacteroidetes(0.9914),c:Bacteroidia(0.5845),o:Bacteroidales(0.3031),f:Porphyromonadaceae(0.1311),g:Paludibacter(0.0215);"	-6.96853	3.49E-18	9.76E-17
OTU177	"d:Bacteria(1.0000),p:Firmicutes(0.9986),c:Clostridia(0.9931),o:Clostridiales(0.9841),f:Ruminococcaceae(0.9687),g:Gemmiger(0.4282);"	-3.49745	7.66E-14	1.84E-12
OTU93	"d:Bacteria(1.0000),p:Firmicutes(0.9978),c:Clostridia(0.9299),o:Clostridiales(0.8337),f:Ruminococcaceae(0.5430),g:Sporobacter(0.0865);"	1.874084	4.85E-13	1.02E-11
OTU22	"d:Bacteria(1.0000),p:Bacteroidetes(0.9922),c:Bacteroidia(0.8348),o:Bacteroidales(0.4658),f:Prevotellaceae(0.1525),g:Paraprevotella(0.0295);"	2.342064	2.15E-12	4.02E-11
OTU3	"d:Bacteria(1.0000),p:Bacteroidetes(0.9914),c:Bacteroidia(0.6471),o:Bacteroidales(0.3394),f:Porphyromonadaceae(0.1525),g:Paludibacter(0.0215);"	2.472215	1.00E-11	1.68E-10
OTU18	"d:Bacteria(1.0000),p:Proteobacteria(0.9329),c:Alphaproteobacteria(0.5845),o:Kiloniellales(0.1942),f:Kiloniellaceae(0.0883),g:Kiloniella(0.0167);"	-3.75285	5.18E-11	7.91E-10
OTU45	"d:Bacteria(0.6389),p:Lentisphaerae(0.0530),c:Lentisphaeria(0.0165),o:Victivallales(0.0062),f:Victivallaceae(0.0015),g:Victivallis(0.0017);"	-3.37142	2.05E-10	2.87E-09
OTU17	"d:Bacteria(1.0000),p:Tenericutes(0.8384),c:Mollicutes(0.5845),o:Acholeplasmatales(0.2305),f:Acholeplasmataceae(0.1097),g:Acholeplasma(0.0191);"	-2.81495	3.26E-09	4.22E-08
OTU90	"d:Bacteria(1.0000),p:Firmicutes(0.9970),c:Clostridia(0.9207),o:Clostridiales(0.8197),f:Eubacteriaceae(0.4275),g:Eubacterium(0.1672);"	1.916869	4.29E-09	5.15E-08
OTU8	"d:Bacteria(1.0000),p:Bacteroidetes(0.9899),c:Sphingobacteriia(0.3342),o:Sphingobacteriales(0.1579),f:Sphingobacteriaceae(0.0668),g:Mucilaginibacter(0.0143);"	2.518286	4.61E-09	5.17E-08
OTU33	"d:Bacteria(1.0000),p:Bacteroidetes(0.7911),c:Bacteroidia(0.0345),o:Bacteroidales(0.0136),f:Porphyromonadaceae(0.0061),g:Barnesiella(0.0043);"	1.956923	2.14E-08	2.25E-07
OTU65	"d:Bacteria(1.0000),p:Firmicutes(0.9914),c:Clostridia(0.8746),o:Clostridiales(0.6422),f:Ruminococcaceae(0.3068),g:Papillibacter(0.0295);"	1.923362	3.55E-08	3.51E-07
OTU202	"d:Bacteria(1.0000),p:Firmicutes(0.9899),c:Clostridia(0.8348),o:Clostridiales(0.4658),f:Ruminococcaceae(0.2263),g:Clostridium_III(0.0215);"	1.491193	1.70E-07	1.58E-06
OTU83	"d:Bacteria(1.0000),p:Bacteroidetes(0.8857),c:Flavobacteriia(0.2113),o:Flavobacteriales(0.0945),f:Flavobacteriaceae(0.0296),g:Riemerella(0.0094);"	2.570017	1.78E-07	1.58E-06
OTU520	"d:Bacteria(1.0000),p:Firmicutes(0.7911),c:Clostridia(0.4593),o:Clostridiales(0.2305),f:Clostridiaceae_1(0.0668),g:Clostridium_sensu_stricto(0.0143);"	-1.75669	3.00E-07	2.50E-06
OTU219	"d:Bacteria(1.0000),p:Firmicutes(0.9802),c:Clostridia(0.8348),o:Clostridiales(0.4658),f:Lachnospiraceae(0.1525),g:Lachnospiracea_incertae_sedis(0.0295);"	-2.1131	3.13E-07	2.50E-06
OTU348	"d:Bacteria(1.0000),p:Firmicutes(0.8857),c:Clostridia(0.7097),o:Clostridiales(0.3775),f:Clostridiaceae_2(0.0883),g:Alkaliphilus(0.0167);"	-2.00052	5.11E-07	3.91E-06
OTU1734	"d:Bacteria(1.0000),p:Proteobacteria(0.3677),c:Deltaproteobacteria(0.1500),o:Bdellovibrionales(0.0673),f:Bdellovibrionaceae(0.0218),g:Vampirovibrio(0.0077);"	-3.46787	8.97E-07	6.56E-06
OTU43	"d:Bacteria(1.0000),p:Firmicutes(0.8147),c:Clostridia(0.5219),o:Clostridiales(0.2668),f:Ruminococcaceae(0.0668),g:Ruminococcus(0.0094);"	-2.49761	1.75E-06	1.23E-05
OTU312	"d:Bacteria(1.0000),p:Firmicutes(0.8620),c:Clostridia(0.6471),o:Clostridiales(0.3394),f:Clostridiaceae_2(0.0883),g:Alkaliphilus(0.0167);"	-1.4519	2.11E-06	1.42E-05
OTU98	"d:Bacteria(1.0000),p:Firmicutes(0.7674),c:Clostridia(0.2725),o:Clostridiales(0.1218),f:Lachnospiraceae(0.0454),g:Anaerostipes(0.0119);"	1.434144	3.34E-06	2.16E-05
OTU20	"d:Bacteria(1.0000),p:Tenericutes(0.9954),c:Mollicutes(0.9299),o:Anaeroplasmatales(0.7305),f:Anaeroplasmataceae(0.5430),g:Anaeroplasma(0.3238);"	-2.73589	4.93E-06	3.07E-05
OTU106	"d:Bacteria(1.0000),p:Firmicutes(0.8620),c:Clostridia(0.6471),o:Clostridiales(0.3394),f:Ruminococcaceae(0.1097),g:Ruminococcus(0.0143);"	1.621601	9.43E-06	5.55E-05
OTU185	"d:Bacteria(1.0000),p:Firmicutes(0.8620),c:Clostridia(0.5845),o:Clostridiales(0.3031),f:Gracilibacteraceae(0.0883),g:Gracilibacter(0.0167);"	1.393046	9.57E-06	5.55E-05
OTU113	"d:Bacteria(1.0000),p:Bacteroidetes(0.9987),c:Bacteroidia(0.9924),o:Bacteroidales(0.9799),f:Prevotellaceae(0.4275),g:Prevotella(0.0637);"	1.789649	2.03E-05	0.000114
OTU122	"d:Bacteria(1.0000),p:Firmicutes(0.9999),c:Bacilli(0.9668),o:Lactobacillales(0.9457),f:Lactobacillaceae(0.9026),g:Lactobacillus(0.8158);"	-1.69073	3.16E-05	0.000171
OTU132	"d:Bacteria(1.0000),p:Firmicutes(0.8620),c:Clostridia(0.6471),o:Clostridiales(0.3394),f:Clostridiaceae_2(0.0668),g:Alkaliphilus(0.0143);"	-2.13043	4.60E-05	0.000242
OTU224	"d:Bacteria(0.2356),p:Proteobacteria(0.0162),c:Alphaproteobacteria(0.0098),o:Rhodospirillales(0.0036),f:Acetobacteraceae(0.0005),g:Stella(0.0009);"	1.474244	7.31E-05	0.000372
OTU153	"d:Bacteria(1.0000),p:Firmicutes(0.9989),c:Clostridia(0.9486),o:Clostridiales(0.8757),f:Lachnospiraceae(0.8051),g:Lachnospiracea_incertae_sedis(0.5751);"	1.44701	7.81E-05	0.000386
OTU64	"d:Bacteria(1.0000),p:Firmicutes(0.7911),c:Erysipelotrichia(0.3967),o:Erysipelotrichales(0.1942),f:Erysipelotrichaceae(0.0883),g:Clostridium_XVIII(0.0167);"	1.60855	8.78E-05	0.000421
OTU159	"d:Bacteria(0.9753),p:Bacteroidetes(0.6840),c:Sphingobacteriia(0.0285),o:Sphingobacteriales(0.0111),f:Sphingobacteriaceae(0.0037),g:Parapedobacter(0.0031);"	2.047345	0.000104	0.000486
OTU32	"d:Bacteria(1.0000),p:Tenericutes(0.9970),c:Mollicutes(0.9449),o:Anaeroplasmatales(0.8057),f:Anaeroplasmataceae(0.6179),g:Anaeroplasma(0.4282);"	-2.05686	0.000149	0.000679
OTU97	"d:Bacteria(1.0000),p:Firmicutes(0.9998),c:Clostridia(0.9814),o:Clostridiales(0.9715),f:Lachnospiraceae(0.9026),g:Clostridium_XlVa(0.8158);"	1.444495	0.000197	0.000859
OTU165	"d:Bacteria(1.0000),p:Bacteroidetes(0.9994),c:Bacteroidia(0.9969),o:Bacteroidales(0.9981),f:Prevotellaceae(0.9770),g:Prevotella(0.9189);"	1.429143	0.000203	0.000859
OTU117	"d:Bacteria(1.0000),p:Firmicutes(0.7674),c:Clostridia(0.3967),o:Clostridiales(0.1942),f:Lachnospiraceae(0.0454),g:Coprococcus(0.0119);"	1.467199	0.000204	0.000859
OTU47	"d:Bacteria(1.0000),p:Firmicutes(0.9566),c:Clostridia(0.8470),o:Clostridiales(0.5099),f:Ruminococcaceae(0.2263),g:Clostridium_IV(0.0167);"	-1.43729	0.000212	0.00087
OTU374	"d:Bacteria(1.0000),p:Bacteroidetes(0.9999),c:Bacteroidia(0.9988),o:Bacteroidales(0.9993),f:Prevotellaceae(0.9898),g:Prevotella(0.9914);"	1.271883	0.000305	0.001219
OTU25	"d:Bacteria(1.0000),p:Proteobacteria(0.3677),c:Deltaproteobacteria(0.1500),o:Bdellovibrionales(0.0673),f:Bdellovibrionaceae(0.0218),g:Vampirovibrio(0.0077);"	-1.72977	0.000392	0.001533
OTU115	"d:Bacteria(0.9753),p:Firmicutes(0.1875),c:Clostridia(0.0345),o:Clostridiales(0.0136),f:Clostridiaceae_1(0.0042),g:Caloramator(0.0034);"	1.484257	0.000557	0.002128
OTU1219	"d:Bacteria(1.0000),p:Proteobacteria(0.5785),c:Deltaproteobacteria(0.2725),o:Bdellovibrionales(0.1218),f:Bdellovibrionaceae(0.0454),g:Vampirovibrio(0.0119);"	1.508311	0.000676	0.002522
OTU34	"d:Bacteria(1.0000),p:Proteobacteria(1.0000),c:Epsilonproteobacteria(1.0000),o:Campylobacterales(1.0000),f:Helicobacteraceae(0.9973),g:Helicobacter(0.9968);"	1.14421	0.000748	0.00273
OTU13	"d:Archaea(1.0000),p:Euryarchaeota(0.9938),c:Thermoplasmata(0.8654),o:Methanomassiliicoccales(0.4658),f:Methanomassiliicoccaceae(0.3068),g:Methanomassiliicoccus(0.0751);"	1.319878	0.000764	0.00273
OTU67	"d:Bacteria(1.0000),p:Firmicutes(0.6840),c:Clostridia(0.3342),o:Clostridiales(0.1579),f:Lachnospiraceae(0.0296),g:Coprococcus(0.0077);"	1.038025	0.001308	0.004578

OTU63	"d:Bacteria(1.0000),p:Firmicutes(0.5785),c:Clostridia(0.0888),o:Clostridiales(0.0400),f:Lachnospiraceae(0.0061),g:Lachnospira(0.0043);"	-1.91691	0.001569	0.00538
OTU76	"d:Bacteria(1.0000),p:Firmicutes(0.9996),c:Clostridia(0.9741),o:Clostridiales(0.9672),f:Lachnospiraceae(0.8778),g:Clostridium_XlVa(0.7126);"	-1.0302	0.001724	0.005792
OTU91	"d:Bacteria(1.0000),p:Firmicutes(0.9946),c:Clostridia(0.5845),o:Clostridiales(0.3031),f:Clostridiaceae_1(0.1525),g:Anaerosporobacter(0.0295);"	-0.99556	0.001813	0.005973
OTU4	"d:Bacteria(1.0000),p:Spirochaetes(0.7911),c:Spirochaetia(0.4593),o:Spirochaetales(0.2305),f:Spirochaetaceae(0.1097),g:Spirochaeta(0.0094);"	-1.54247	0.002002	0.006467
OTU2828	"d:Bacteria(1.0000),p:Firmicutes(0.9984),c:Clostridia(0.9937),o:Clostridiales(0.9926),f:Ruminococcaceae(0.9847),g:Faecalibacterium(0.8158);"	-1.01428	0.002349	0.007445
OTU428	"d:Bacteria(1.0000),p:Firmicutes(0.9899),c:Clostridia(0.7723),o:Clostridiales(0.4216),f:Clostridiaceae_1(0.1311),g:Clostridium_sensu_stricto(0.0215);"	-0.94408	0.002509	0.007804

Table S3. Results from exact tests showing OTUs with significantly different occurrence in Odobullu relative to Kokosa, including full taxonomic assignment, log-fold difference in abundance (logFC), test p-value and false discovery rate (FDR).

OTU#	Taxonomy	logFC	PValue	FDR
OTU835	"d:Bacteria(1.0000),p:Bacteroidetes(0.9914),c:Bacteroidia(0.5845),o:Bacteroidales(0.3031),f:Porphyromonadaceae(0.1311),g:Paludibacter(0.0215);"	-9.33901	8.83E-26	1.48E-23
OTU21	"d:Bacteria(1.0000),p:Proteobacteria(0.9987),c:Betaproteobacteria(0.9115),o:Rhodocyclales(0.5981),f:Rhodocyclaceae(0.4275),g:Denitratisoma(0.1672);"	-3.94013	4.70E-24	3.95E-22
OTU179	"d:Bacteria(1.0000),p:Firmicutes(0.9962),c:Clostridia(0.9023),o:Clostridiales(0.7746),f:Ruminococcaceae(0.5055),g:Sporobacter(0.0751);"	2.187449	3.96E-16	2.22E-14
OTU202	"d:Bacteria(1.0000),p:Firmicutes(0.9899),c:Clostridia(0.8348),o:Clostridiales(0.4658),f:Ruminococcaceae(0.2263),g:Clostridium_III(0.0215);"	1.672245	6.33E-09	2.51E-07
OTU18	"d:Bacteria(1.0000),p:Proteobacteria(0.9329),c:Alphaproteobacteria(0.5845),o:Kiloniellales(0.1942),f:Kiloniellaceae(0.0883),g:Kiloniella(0.0167);"	-3.1887	8.60E-09	2.51E-07
OTU25	"d:Bacteria(1.0000),p:Proteobacteria(0.3677),c:Deltaproteobacteria(0.1500),o:Bdellovibrionales(0.0673),f:Bdellovibrionaceae(0.0218),g:Vampirovibrio(0.0077);"	-3.03627	8.95E-09	2.51E-07
OTU177	"d:Bacteria(1.0000),p:Firmicutes(0.9986),c:Clostridia(0.9931),o:Clostridiales(0.9841),f:Ruminococcaceae(0.9687),g:Gemmiger(0.4282);"	-2.39453	5.02E-08	1.20E-06
OTU19	"d:Bacteria(1.0000),p:Bacteroidetes(0.9988),c:Bacteroidia(0.8654),o:Bacteroidales(0.5981),f:Porphyromonadaceae(0.4275),g:Parabacteroides(0.0409);"	-2.37059	2.02E-07	4.25E-06
OTU32	"d:Bacteria(1.0000),p:Tenericutes(0.9970),c:Mollicutes(0.9449),o:Anaeroplasmatales(0.8057),f:Anaeroplasmataceae(0.6179),g:Anaeroplasma(0.4282);"	-2.93843	2.89E-07	5.39E-06
OTU73	"d:Bacteria(1.0000),p:Proteobacteria(0.7674),c:Alphaproteobacteria(0.2113),o:Kiloniellales(0.0400),f:Kiloniellaceae(0.0140),g:Kiloniella(0.0060);"	2.600434	5.70E-07	9.58E-06
OTU126	"d:Bacteria(1.0000),p:Firmicutes(0.7911),c:Clostridia(0.4593),o:Clostridiales(0.2305),f:Clostridiaceae_4(0.0296),g:Thermotalea(0.0094);"	-1.9175	1.35E-06	2.06E-05
OTU348	"d:Bacteria(1.0000),p:Firmicutes(0.8857),c:Clostridia(0.7097),o:Clostridiales(0.3775),f:Clostridiaceae_2(0.0883),g:Alkaliphilus(0.0167);"	-1.90455	1.66E-06	2.33E-05
OTU235	"d:Bacteria(1.0000),p:Actinobacteria(0.9914),c:Actinobacteria(0.8562),o:Coriobacteriales(0.5540),f:Coriobacteriaceae(0.3872),g:Slackia(0.0295);"	1.387921	2.61E-06	3.37E-05
OTU52	"d:Bacteria(1.0000),p:Firmicutes(0.7674),c:Clostridia(0.3967),o:Clostridiales(0.1218),f:Ruminococcaceae(0.0454),g:Ruminococcus(0.0094);"	2.579006	3.71E-06	4.46E-05
OTU85	"d:Bacteria(1.0000),p:Bacteroidetes(0.8384),c:Bacteroidia(0.3342),o:Bacteroidales(0.1579),f:Bacteroidaceae(0.0042),g:Bacteroides(0.0034);"	2.283826	1.45E-05	0.000162
OTU42	"d:Bacteria(0.7884),p:Proteobacteria(0.1203),c:Deltaproteobacteria(0.0255),o:Bdellovibrionales(0.0099),f:Bdellovibrionaceae(0.0031),g:Vampirovibrio(0.0027);"	-2.76106	2.32E-05	0.000244
OTU4	"d:Bacteria(1.0000),p:Spirochaetes(0.7911),c:Spirochaetia(0.4593),o:Spirochaetales(0.2305),f:Spirochaetaceae(0.1097),g:Spirochaeta(0.0094);"	-2.11368	4.33E-05	0.000425
OTU143	"d:Bacteria(1.0000),p:Firmicutes(0.9994),c:Clostridia(0.9704),o:Clostridiales(0.9597),f:Lachnospiraceae(0.8051),g:Clostridium_XlVa(0.6095);"	1.157632	4.55E-05	0.000425
OTU62	"d:Bacteria(1.0000),p:Bacteroidetes(0.9329),c:Bacteroidia(0.8348),o:Bacteroidales(0.4658),f:Prevotellaceae(0.1097),g:Alloprevotella(0.0143);"	-1.91835	5.71E-05	0.000505
OTU217	"d:Bacteria(1.0000),p:Firmicutes(0.3677),c:Clostridia(0.1500),o:Clostridiales(0.0673),f:Ruminococcaceae(0.0140),g:Ruminococcus(0.0060);"	-2.31671	9.38E-05	0.000788
OTU181	"d:Bacteria(1.0000),p:Firmicutes(0.9983),c:Clostridia(0.9929),o:Clostridiales(0.9841),f:Lachnospiraceae(0.9274),g:Pseudobutyrivibrio(0.7126);"	1.281214	0.000132	0.001056
OTU224	"d:Bacteria(0.2356),p:Proteobacteria(0.0162),c:Alphaproteobacteria(0.0098),o:Rhodospirillales(0.0036),f:Acetobacteraceae(0.0005),g:Stella(0.0009);"	1.411335	0.000175	0.001335
OTU108	"d:Bacteria(1.0000),p:Firmicutes(0.9992),c:Clostridia(0.9486),o:Clostridiales(0.8757),f:Ruminococcaceae(0.6553),g:Papillibacter(0.1150);"	-1.5772	0.000188	0.001373
OTU30	"d:Bacteria(1.0000),p:Firmicutes(0.9992),c:Erysipelotrichia(0.9595),o:Erysipelotrichales(0.9177),f:Erysipelotrichaceae(0.8861),g:Clostridium_XVIII(0.3760);"	-1.45065	0.000271	0.0019
OTU47	"d:Bacteria(1.0000),p:Firmicutes(0.9566),c:Clostridia(0.8470),o:Clostridiales(0.5099),f:Ruminococcaceae(0.2263),g:Clostridium_IV(0.0167);"	-1.36896	0.000423	0.00284
OTU93	"d:Bacteria(1.0000),p:Firmicutes(0.9978),c:Clostridia(0.9299),o:Clostridiales(0.8337),f:Ruminococcaceae(0.5430),g:Sporobacter(0.0865);"	0.924848	0.000496	0.003202
OTU99	"d:Bacteria(1.0000),p:Actinobacteria(0.9938),c:Actinobacteria(0.8931),o:Coriobacteriales(0.7305),f:Coriobacteriaceae(0.5430),g:Slackia(0.0751);"	1.143596	0.000528	0.003228
OTU76	"d:Bacteria(1.0000),p:Firmicutes(0.9996),c:Clostridia(0.9741),o:Clostridiales(0.9672),f:Lachnospiraceae(0.8778),g:Clostridium_XlVa(0.7126);"	-1.14841	0.000545	0.003228
OTU7	"d:Bacteria(1.0000),p:Firmicutes(0.9991),c:Clostridia(0.9964),o:Clostridiales(0.9975),f:Ruminococcaceae(0.9973),g:Faecalibacterium(0.9829);"	-0.90103	0.000569	0.003228
OTU133	"d:Bacteria(1.0000),p:Firmicutes(0.6840),c:Clostridia(0.3342),o:Clostridiales(0.1218),f:Ruminococcaceae(0.0296),g:Intestinimonas(0.0077);"	-1.63768	0.000581	0.003228
OTU135	"d:Bacteria(1.0000),p:Tenericutes(0.4731),c:Mollicutes(0.2113),o:Acholeplasmatales(0.0945),f:Acholeplasmataceae(0.0296),g:Acholeplasma(0.0094);"	-2.16688	0.000596	0.003228
OTU26	"d:Bacteria(1.0000),p:Tenericutes(0.4731),c:Mollicutes(0.2113),o:Anaeroplasmatales(0.0945),f:Anaeroplasmataceae(0.0296),g:Asteroleplasma(0.0094);"	-1.85377	0.000655	0.003439
OTU97	"d:Bacteria(1.0000),p:Firmicutes(0.9998),c:Clostridia(0.9814),o:Clostridiales(0.9715),f:Lachnospiraceae(0.9026),g:Clostridium_XlVa(0.8158);"	1.322178	0.000761	0.003876
OTU104	"d:Bacteria(1.0000),p:Firmicutes(0.5785),c:Clostridia(0.2725),o:Clostridiales(0.1218),f:Ruminococcaceae(0.0218),g:Intestinimonas(0.0060);"	1.499128	0.001313	0.00649
OTU100	"d:Bacteria(1.0000),p:Firmicutes(0.9946),c:Clostridia(0.9115),o:Clostridiales(0.8057),f:Ruminococcaceae(0.3872),g:Papillibacter(0.0523);"	0.782397	0.001519	0.007289
OTU50	"d:Bacteria(1.0000),p:Actinobacteria(1.0000),c:Actinobacteria(1.0000),o:Bifidobacteriales(0.9966),f:Bifidobacteriaceae(0.9970),g:Bifidobacterium(0.9871);"	-1.9292	0.001634	0.007626
OTU146	"d:Bacteria(1.0000),p:Firmicutes(0.7911),c:Negativicutes(0.2725),o:Selenomonadales(0.1218),f:Veillonellaceae(0.0454),g:Dendrosporobacter(0.0119);"	-0.96379	0.002191	0.009949

Table S4. Unpaired t-tests comparing GI microbiota diversity between Bale monkey populations

Comparison	Metric	P-value
Afursa vs. Kokosa	Shannon entropy	0.241
Afursa vs. Odobullu	Shannon entropy	0.055
Kokosa vs Odobullu	Shannon entropy	0.001
Afursa vs. Kokosa	#OTUs	<0.001
Afursa vs. Odobullu	#OTUs	0.647
Kokosa vs Odobullu	#OTUs	0.003
Afursa vs. Kokosa	Chao1	0.052
Afursa vs. Odobullu	Chao1	0.09
Kokosa vs Odobullu	Chao1	0.114

Table S5. Results from exact tests showing OTUs with significantly different occurrence in the wet relative to the dry season in Afursa, including full taxonomic assignment, log-fold difference in abundance (logFC), test p-value and false discovery rate (FDR).

OTU#	Taxonomy	logFC	PValue	FDR
OTU132	"d:Bacteria(1.0000),p:Firmicutes(0.8620),c:Clostridia(0.6471),o:Clostridiales(0.3394),f:Clostridiaceae_2(0.0668),g:Alkaliphilus(0.0143);"	2.982014	2.07E-10	3.70E-08
OTU228	"d:Bacteria(1.0000),p:Firmicutes(0.9987),c:Clostridia(0.9522),o:Clostridiales(0.8897),f:Lachnospiraceae(0.7302),g:Anaerostipes(0.5751);"	-2.53138	8.65E-09	7.75E-07
OTU900	"d:Bacteria(1.0000),p:Firmicutes(0.9998),c:Clostridia(0.9981),o:Clostridiales(0.9956),f:Ruminococcaceae(0.9898),g:Faecalibacterium(0.8158);"	-1.67758	1.67E-07	9.97E-06
OTU201	"d:Bacteria(1.0000),p:Firmicutes(0.8384),c:Clostridia(0.5845),o:Clostridiales(0.3031),f:Ruminococcaceae(0.1311),g:Ruminococcus(0.0191);"	1.629095	5.40E-06	0.000242
OTU131	"d:Bacteria(1.0000),p:Firmicutes(0.9987),c:Clostridia(0.9950),o:Clostridiales(0.9960),f:Ruminococcaceae(0.9919),g:Faecalibacterium(0.9701);"	-1.30778	1.26E-05	0.00045
OTU439	"d:Bacteria(1.0000),p:Bacteroidetes(1.0000),c:Bacteroidia(1.0000),o:Bacteroidales(1.0000),f:Bacteroidaceae(0.9929),g:Bacteroides(0.9968);"	1.58799	2.21E-05	0.00066
OTU7	"d:Bacteria(1.0000),p:Firmicutes(0.9991),c:Clostridia(0.9964),o:Clostridiales(0.9975),f:Ruminococcaceae(0.9973),g:Faecalibacterium(0.9829);"	-1.06418	5.62E-05	0.001437
OTU61	"d:Bacteria(1.0000),p:Proteobacteria(0.4731),c:Deltaproteobacteria(0.2113),o:Bdellovibrionales(0.0945),f:Bdellovibrionaceae(0.0296),g:Vampirovibrio(0.0094);"	1.51765	9.05E-05	0.001852
OTU30	"d:Bacteria(1.0000),p:Firmicutes(0.9992),c:Erysipelotrichia(0.9595),o:Erysipelotrichales(0.9177),f:Erysipelotrichaceae(0.8861),g:Clostridium_XVIII(0.3760);"	-1.19747	9.31E-05	0.001852
OTU193	"d:Bacteria(1.0000),p:Firmicutes(0.9998),c:Clostridia(0.9939),o:Clostridiales(0.9947),f:Lachnospiraceae(0.9274),g:Blautia(0.7814);"	-0.96393	0.000107	0.001913
OTU129	"d:Bacteria(1.0000),p:Firmicutes(0.7674),c:Clostridia(0.3967),o:Clostridiales(0.1942),f:Clostridiaceae_3(0.0668),g:Brassicibacter(0.0143);"	1.536502	0.000155	0.00246
OTU136	"d:Bacteria(1.0000),p:Tenericutes(0.2622),c:Mollicutes(0.0888),o:Entomoplasmatales(0.0400),f:Spiroplasmataceae(0.0140),g:Spiroplasma(0.0060);"	1.567613	0.000165	0.00246
OTU237	"d:Bacteria(1.0000),p:Tenericutes(0.7674),c:Mollicutes(0.3967),o:Entomoplasmatales(0.1942),f:Spiroplasmataceae(0.0883),g:Spiroplasma(0.0167);"	1.331023	0.000246	0.003393
OTU178	"d:Bacteria(1.0000),p:Firmicutes(0.9998),c:Clostridia(0.9559),o:Clostridiales(0.9037),f:Lachnospiraceae(0.8778),g:Lachnospiracea_incertae_sedis(0.7126);"	-1.31597	0.000297	0.003792
OTU73	"d:Bacteria(1.0000),p:Proteobacteria(0.7674),c:Alphaproteobacteria(0.2113),o:Kiloniellales(0.0400),f:Kiloniellaceae(0.0140),g:Kiloniella(0.0060);"	-2.19012	0.000485	0.005783
OTU520	"d:Bacteria(1.0000),p:Firmicutes(0.7911),c:Clostridia(0.4593),o:Clostridiales(0.2305),f:Clostridiaceae_1(0.0668),g:Clostridium_sensu_stricto(0.0143);"	1.113974	0.000562	0.00629
OTU255	"d:Bacteria(1.0000),p:Firmicutes(0.9990),c:Clostridia(0.9668),o:Clostridiales(0.9457),f:Ruminococcaceae(0.8425),g:Ruminococcus(0.6438);"	-1.47491	0.000607	0.006387
OTU147	"d:Bacteria(1.0000),p:Firmicutes(0.9988),c:Clostridia(0.9559),o:Clostridiales(0.9037),f:Lachnospiraceae(0.7302),g:Clostridium_XlVa(0.4804);"	1.277595	0.000993	0.009876

Table S6. Results from exact tests showing OTUs with significantly different occurrence in the wet relative to the dry season in Kokosa, including full taxonomic assignment, log-fold difference in abundance (logFC), test p-value and false discovery rate (FDR).

OTU#	Taxonomy	logFC	PValue	FDR
OTU107	"d:Bacteria(1.0000),p:Firmicutes(0.9983),c:Clostridia(0.9924),o:Clostridiales(0.9799),f:Lachnospiraceae(0.9191),g:Anaerostipes(0.8846);"	-5.36778	5.73E-22	9.23E-20
OTU38	"d:Bacteria(1.0000),p:Firmicutes(0.9984),c:Negativicutes(0.9935),o:Selenomonadales(0.9904),f:Veillonellaceae(0.9867),g:Dialister(0.9701);"	-3.52886	3.85E-13	3.10E-11
OTU58	"d:Bacteria(1.0000),p:Firmicutes(1.0000),c:Clostridia(0.9961),o:Clostridiales(0.9970),f:Lachnospiraceae(0.9878),g:Blautia(0.9743);"	-2.6896	1.14E-11	6.11E-10
OTU108	"d:Bacteria(1.0000),p:Firmicutes(0.9992),c:Clostridia(0.9486),o:Clostridiales(0.8757),f:Ruminococcaceae(0.6553),g:Papillibacter(0.1150);"	-2.97752	2.00E-10	6.91E-09
OTU69	"d:Bacteria(1.0000),p:Firmicutes(0.9991),c:Clostridia(0.9668),o:Clostridiales(0.9457),f:Ruminococcaceae(0.4677),g:Butyricicoccus(0.2194);"	-3.37911	2.15E-10	6.91E-09
OTU13	"d:Archaea(1.0000),p:Euryarchaeota(0.9938),c:Thermoplasmata(0.8654),o:Methanomassiliicoccales(0.4658),f:Methanomassiliicoccaceae(0.3068),g:Methanomassiliicoccus(0.0751);"	2.53461	5.12E-10	1.37E-08
OTU30	"d:Bacteria(1.0000),p:Firmicutes(0.9992),c:Erysipelotrichia(0.9595),o:Erysipelotrichales(0.9177),f:Erysipelotrichaceae(0.8861),g:Clostridium_XVIII(0.3760);"	-3.29676	9.84E-10	2.26E-08
OTU193	"d:Bacteria(1.0000),p:Firmicutes(0.9998),c:Clostridia(0.9939),o:Clostridiales(0.9947),f:Lachnospiraceae(0.9274),g:Blautia(0.7814);"	-2.3011	1.30E-09	2.61E-08
OTU130	"d:Bacteria(1.0000),p:Firmicutes(0.9984),c:Clostridia(0.9814),o:Clostridiales(0.9715),f:Lachnospiraceae(0.9357),g:Lachnospiracea_incertae_sedis(0.9189);"	-2.57611	1.99E-09	3.57E-08
OTU109	"d:Bacteria(1.0000),p:Firmicutes(1.0000),c:Clostridia(0.9941),o:Clostridiales(0.9952),f:Lachnospiraceae(0.9837),g:Dorea(0.9701);"	-2.51858	3.03E-09	4.88E-08
OTU187	"d:Bacteria(1.0000),p:Firmicutes(0.9988),c:Clostridia(0.9948),o:Clostridiales(0.9958),f:Lachnospiraceae(0.9908),g:Coprococcus(0.9871);"	-2.83913	5.15E-09	7.02E-08
OTU61	"d:Bacteria(1.0000),p:Proteobacteria(0.4731),c:Deltaproteobacteria(0.2113),o:Bdellovibrionales(0.0945),f:Bdellovibrionaceae(0.0296),g:Vampirovibrio(0.0094);"	3.041131	5.24E-09	7.02E-08
OTU118	"d:Bacteria(1.0000),p:Firmicutes(0.9999),c:Clostridia(0.9990),o:Clostridiales(0.9994),f:Ruminococcaceae(0.9992),g:Subdoligranulum(0.6438);"	-2.20724	6.28E-09	7.77E-08
OTU166	"d:Bacteria(1.0000),p:Firmicutes(1.0000),c:Clostridia(0.9958),o:Clostridiales(0.9968),f:Lachnospiraceae(0.9770),g:Blautia(0.9743);"	-2.34775	1.02E-08	1.17E-07
OTU154	"d:Bacteria(1.0000),p:Firmicutes(0.2622),c:Clostridia(0.0888),o:Clostridiales(0.0400),f:Clostridiales_Incertae_Sedis_XII(0.0140),g:Guggenheimella(0.0060);"	-2.66663	1.72E-08	1.85E-07
OTU23	"d:Bacteria(1.0000),p:Bacteroidetes(0.9996),c:Bacteroidia(0.9974),o:Bacteroidales(0.9986),f:Prevotellaceae(0.9687),g:Prevotella(0.9722);"	-2.40044	5.90E-08	5.89E-07
OTU139	"d:Bacteria(1.0000),p:Firmicutes(0.9899),c:Clostridia(0.8654),o:Clostridiales(0.5099),f:Ruminococcaceae(0.3470),g:Intestinimonas(0.0523);"	2.345301	6.22E-08	5.89E-07
OTU91	"d:Bacteria(1.0000),p:Firmicutes(0.9946),c:Clostridia(0.5845),o:Clostridiales(0.3031),f:Clostridiaceae_1(0.1525),g:Anaerosporobacter(0.0295);"	-2.06677	7.08E-08	6.33E-07
OTU8	"d:Bacteria(1.0000),p:Bacteroidetes(0.9899),c:Sphingobacteriia(0.3342),o:Sphingobacteriales(0.1579),f:Sphingobacteriaceae(0.0668),g:Mucilaginibacter(0.0143);"	2.878031	8.64E-08	7.32E-07
OTU230	"d:Bacteria(1.0000),p:Firmicutes(0.9998),c:Clostridia(0.9933),o:Clostridiales(0.9883),f:Lachnospiraceae(0.9109),g:Blautia(0.6438);"	-1.7607	9.39E-08	7.56E-07
OTU36	"d:Bacteria(1.0000),p:Bacteroidetes(0.9995),c:Bacteroidia(0.9973),o:Bacteroidales(0.9985),f:Prevotellaceae(0.9687),g:Prevotella(0.9722);"	-1.94797	2.08E-06	1.60E-05
OTU374	"d:Bacteria(1.0000),p:Bacteroidetes(0.9999),c:Bacteroidia(0.9988),o:Bacteroidales(0.9993),f:Prevotellaceae(0.9898),g:Prevotella(0.9914);"	-2.24043	2.36E-06	1.73E-05
OTU900	"d:Bacteria(1.0000),p:Firmicutes(0.9998),c:Clostridia(0.9981),o:Clostridiales(0.9956),f:Ruminococcaceae(0.9898),g:Faecalibacterium(0.8158);"	-1.95963	3.66E-06	2.56E-05
OTU21	"d:Bacteria(1.0000),p:Proteobacteria(0.9987),c:Betaproteobacteria(0.9115),o:Rhodocyclales(0.5981),f:Rhodocyclaceae(0.4275),g:Denitratisoma(0.1672);"	1.635134	6.05E-06	4.06E-05
OTU3	"d:Bacteria(1.0000),p:Bacteroidetes(0.9914),c:Bacteroidia(0.6471),o:Bacteroidales(0.3394),f:Porphyromonadaceae(0.1525),g:Paludibacter(0.0215);"	1.747607	8.55E-06	5.51E-05
OTU7	"d:Bacteria(1.0000),p:Firmicutes(0.9991),c:Clostridia(0.9964),o:Clostridiales(0.9975),f:Ruminococcaceae(0.9973),g:Faecalibacterium(0.9829);"	-1.39752	1.36E-05	8.45E-05
OTU146	"d:Bacteria(1.0000),p:Firmicutes(0.7911),c:Negativicutes(0.2725),o:Selenomonadales(0.1218),f:Veillonellaceae(0.0454),g:Dendrosporobacter(0.0119);"	-1.71168	1.47E-05	8.77E-05
OTU34	"d:Bacteria(1.0000),p:Proteobacteria(1.0000),c:Epsilonproteobacteria(1.0000),o:Campylobacterales(1.0000),f:Helicobacteraceae(0.9973),g:Helicobacter(0.9968);"	-2.04445	2.01E-05	0.000116
OTU83	"d:Bacteria(1.0000),p:Bacteroidetes(0.8857),c:Flavobacteriia(0.2113),o:Flavobacteriales(0.0945),f:Flavobacteriaceae(0.0296),g:Riemerella(0.0094);"	2.367879	2.73E-05	0.000149
OTU5	"d:Bacteria(1.0000),p:Bacteroidetes(0.9988),c:Bacteroidia(0.9886),o:Bacteroidales(0.9757),f:Prevotellaceae(0.5055),g:Prevotella(0.1672);"	1.978324	2.78E-05	0.000149
OTU56	"d:Bacteria(1.0000),p:Bacteroidetes(0.9930),c:Bacteroidia(0.8348),o:Bacteroidales(0.4658),f:Porphyromonadaceae(0.3068),g:Parabacteroides(0.0215);"	1.935771	4.76E-05	0.000247
OTU263	"d:Bacteria(1.0000),p:Firmicutes(0.6840),c:Clostridia(0.3342),o:Clostridiales(0.1579),f:Clostridiaceae_4(0.0296),g:Thermotalea(0.0094);"	1.8969	0.000139	0.000687
OTU155	"d:Bacteria(1.0000),p:Firmicutes(0.9899),c:Clostridia(0.8654),o:Clostridiales(0.4216),f:Ruminococcaceae(0.2263),g:Intestinimonas(0.0409);"	1.71576	0.000141	0.000687
OTU18	"d:Bacteria(1.0000),p:Proteobacteria(0.9329),c:Alphaproteobacteria(0.5845),o:Kiloniellales(0.1942),f:Kiloniellaceae(0.0883),g:Kiloniella(0.0167);"	1.789523	0.000158	0.000734
OTU98	"d:Bacteria(1.0000),p:Firmicutes(0.7674),c:Clostridia(0.2725),o:Clostridiales(0.1218),f:Lachnospiraceae(0.0454),g:Anaerostipes(0.0119);"	1.145138	0.000159	0.000734
OTU86	"d:Bacteria(1.0000),p:Proteobacteria(0.5785),c:Deltaproteobacteria(0.2725),o:Bdellovibrionales(0.0945),f:Bdellovibrionaceae(0.0296),g:Vampirovibrio(0.0094);"	2.001259	0.000195	0.00087
OTU2	"d:Bacteria(1.0000),p:Firmicutes(0.9987),c:Clostridia(0.9943),o:Clostridiales(0.9954),f:Clostridiaceae_1(0.8778),g:Clostridium_sensu_stricto(0.7126);"	-2.35865	0.000228	0.000993
OTU25	"d:Bacteria(1.0000),p:Proteobacteria(0.3677),c:Deltaproteobacteria(0.1500),o:Bdellovibrionales(0.0673),f:Bdellovibrionaceae(0.0218),g:Vampirovibrio(0.0077);"	-2.07747	0.000254	0.001076
OTU87	"d:Bacteria(1.0000),p:Bacteroidetes(0.9922),c:Bacteroidia(0.8348),o:Bacteroidales(0.4658),f:Porphyromonadaceae(0.3068),g:Barnesiella(0.0215);"	1.327237	0.00028	0.001155
OTU14	"d:Bacteria(1.0000),p:Firmicutes(0.6840),c:Clostridia(0.3342),o:Clostridiales(0.1579),f:Clostridiaceae_4(0.0296),g:Thermotalea(0.0094);"	-2.18178	0.00037	0.001489
OTU312	"d:Bacteria(1.0000),p:Firmicutes(0.8620),c:Clostridia(0.6471),o:Clostridiales(0.3394),f:Clostridiaceae_2(0.0883),g:Alkaliphilus(0.0167);"	-1.31818	0.000408	0.001603
OTU66	"d:Bacteria(1.0000),p:Actinobacteria(0.2622),c:Actinobacteria(0.0888),o:Actinomycetales(0.0400),f:Nocardiaceae(0.0140),g:Rhodococcus(0.0060);"	-2.11433	0.000497	0.001906
OTU1219	"d:Bacteria(1.0000),p:Proteobacteria(0.5785),c:Deltaproteobacteria(0.2725),o:Bdellovibrionales(0.1218),f:Bdellovibrionaceae(0.0454),g:Vampirovibrio(0.0119);"	1.892635	0.000826	0.003094
OTU116	"d:Bacteria(1.0000),p:Firmicutes(1.0000),c:Clostridia(0.9950),o:Clostridiales(0.9960),f:Lachnospiraceae(0.9888),g:Roseburia(0.9786);"	-1.21086	0.000907	0.003317
OTU168	"d:Bacteria(1.0000),p:Bacteroidetes(0.9566),c:Bacteroidia(0.5219),o:Bacteroidales(0.2668),f:Porphyromonadaceae(0.1311),g:Barnesiella(0.0143);"	-1.5393	0.001251	0.004477
OTU428	"d:Bacteria(1.0000),p:Firmicutes(0.9899),c:Clostridia(0.7723),o:Clostridiales(0.4216),f:Clostridiaceae_1(0.1311),g:Clostridium_sensu_stricto(0.0215);"	-1.29008	0.001457	0.0051
OTU128	"d:Bacteria(1.0000),p:Bacteroidetes(0.9984),c:Bacteroidia(0.9937),o:Bacteroidales(0.9926),f:Prevotellaceae(0.6553),g:Prevotella(0.3760);"	-1.5948	0.001638	0.00561

Table S7. Results from exact tests showing OTUs with significantly different occurrence in the wet relative to the dry season in Odobullu, including full taxonomic assignment, log-fold difference in abundance (logFC), test p-value and false discovery rate (FDR).

OTU#	Taxonomy	logFC	PValue	FDR
OTU409	"d:Bacteria(1.0000),p:Firmicutes(0.9978),c:Bacilli(0.8348),o:Lactobacillales(0.4658),f:Lactobacillaceae(0.3068),g:Lactobacillus(0.0751);"	-6.75492	4.35E-11	7.75E-09
OTU478	"d:Bacteria(1.0000),p:Firmicutes(0.9987),c:Clostridia(0.9922),o:Clostridiales(0.9778),f:Lachnospiraceae(0.9191),g:Clostridium_XlVb(0.7126);"	-2.80529	4.23E-06	0.000256
OTU47	"d:Bacteria(1.0000),p:Firmicutes(0.9566),c:Clostridia(0.8470),o:Clostridiales(0.5099),f:Ruminococcaceae(0.2263),g:Clostridium_IV(0.0167);"	2.822137	4.32E-06	0.000256
OTU111	"d:Bacteria(1.0000),p:Firmicutes(0.9998),c:Clostridia(0.9922),o:Clostridiales(0.9778),f:Lachnospiraceae(0.8943),g:Clostridium_XlVa(0.6095);"	-2.4589	6.72E-06	0.000299
OTU363	"d:Bacteria(1.0000),p:Firmicutes(0.9998),c:Clostridia(0.9850),o:Clostridiales(0.9736),f:Lachnospiraceae(0.8778),g:Clostridium_XlVa(0.7126);"	-3.5429	1.52E-05	0.000542
OTU179	"d:Bacteria(1.0000),p:Firmicutes(0.9962),c:Clostridia(0.9023),o:Clostridiales(0.7746),f:Ruminococcaceae(0.5055),g:Sporobacter(0.0751);"	-2.14578	2.07E-05	0.000615
OTU51	"d:Bacteria(1.0000),p:Bacteroidetes(0.9988),c:Bacteroidia(0.9922),o:Bacteroidales(0.9778),f:Prevotellaceae(0.4677),g:Prevotella(0.0523);"	2.142334	3.92E-05	0.000996
OTU153	"d:Bacteria(1.0000),p:Firmicutes(0.9989),c:Clostridia(0.9486),o:Clostridiales(0.8757),f:Lachnospiraceae(0.8051),g:Lachnospiracea_incertae_sedis(0.5751);"	-2.77271	5.10E-05	0.001134
OTU48	"d:Bacteria(1.0000),p:Actinobacteria(1.0000),c:Actinobacteria(1.0000),o:Actinomycetales(1.0000),f:Micrococcaceae(0.9867),g:Arthrobacter(0.8846);"	-3.80688	6.08E-05	0.001203
OTU97	"d:Bacteria(1.0000),p:Firmicutes(0.9998),c:Clostridia(0.9814),o:Clostridiales(0.9715),f:Lachnospiraceae(0.9026),g:Clostridium_XlVa(0.8158);"	-2.97556	7.94E-05	0.001414
OTU150	"d:Bacteria(1.0000),p:Firmicutes(0.9970),c:Clostridia(0.9391),o:Clostridiales(0.8477),f:Ruminococcaceae(0.5804),g:Ruminococcus(0.3238);"	-3.0826	8.94E-05	0.001446
OTU87	"d:Bacteria(1.0000),p:Bacteroidetes(0.9922),c:Bacteroidia(0.8348),o:Bacteroidales(0.4658),f:Porphyromonadaceae(0.3068),g:Barnesiella(0.0215);"	2.079264	0.000145	0.002147
OTU170	"d:Bacteria(1.0000),p:Proteobacteria(0.6840),c:Alphaproteobacteria(0.3342),o:Rhodospirillales(0.1218),f:Rhodospirillaceae(0.0454),g:Insolitispirillum(0.0043);"	-2.94798	0.000442	0.005874
OTU123	"d:Bacteria(1.0000),p:Firmicutes(0.9914),c:Clostridia(0.8746),o:Clostridiales(0.5540),f:Ruminococcaceae(0.3068),g:Pseudoflavonifractor(0.0215);"	2.324612	0.000462	0.005874

Table S8. Results from exact tests showing OTUs with significantly different occurrence in grivets/vervets relative to Bale monkeys, including full taxonomic assignment, log-fold difference in abundance (logFC), test p-value and false discovery rate (FDR).

OTU#	Taxonomy	logFC	PValue	FDR
OTU87	"d:Bacteria(1.0000),p:Bacteroidetes(0.9922),c:Bacteroidia(0.8348),o:Bacteroidales(0.4658),f:Porphyromonadaceae(0.3068),g:Barnesiella(0.0215);"	-7.55043	6.68E-66	9.95E-64
OTU5	"d:Bacteria(1.0000),p:Bacteroidetes(0.9988),c:Bacteroidia(0.9886),o:Bacteroidales(0.9757),f:Prevotellaceae(0.5055),g:Prevotella(0.1672);"	-7.92719	1.58E-55	1.18E-53
OTU12	"d:Bacteria(1.0000),p:Bacteroidetes(0.9985),c:Bacteroidia(0.9935),o:Bacteroidales(0.9904),f:Prevotellaceae(0.6928),g:Prevotella(0.4804);"	-8.27175	1.41E-53	6.98E-52
OTU2	"d:Bacteria(1.0000),p:Firmicutes(0.9987),c:Clostridia(0.9943),o:Clostridiales(0.9954),f:Clostridiaceae_1(0.8778),g:Clostridium_sensu_stricto(0.7126);"	-7.19974	3.87E-41	1.44E-39
OTU165	"d:Bacteria(1.0000),p:Bacteroidetes(0.9994),c:Bacteroidia(0.9969),o:Bacteroidales(0.9981),f:Prevotellaceae(0.9770),g:Prevotella(0.9189);"	-5.42909	1.02E-33	3.03E-32
OTU109	"d:Bacteria(1.0000),p:Firmicutes(1.0000),c:Clostridia(0.9941),o:Clostridiales(0.9952),f:Lachnospiraceae(0.9837),g:Dorea(0.9701);"	2.474253	1.94E-32	4.81E-31
OTU485	"d:Bacteria(1.0000),p:Firmicutes(0.9991),c:Clostridia(0.9559),o:Clostridiales(0.9037),f:Lachnospiraceae(0.6928),g:Clostridium_XlVa(0.3760);"	1.815132	3.35E-28	7.14E-27
OTU79	"d:Bacteria(1.0000),p:Firmicutes(0.9914),c:Clostridia(0.8562),o:Clostridiales(0.5540),f:Ruminococcaceae(0.2263),g:Papillibacter(0.0295);"	1.921816	1.56E-27	2.91E-26
OTU247	"d:Bacteria(1.0000),p:Firmicutes(0.6840),c:Clostridia(0.2113),o:Clostridiales(0.0945),f:Lachnospiraceae(0.0296),g:Clostridium_XlVa(0.0077);"	-4.50795	3.54E-26	5.86E-25
OTU58	"d:Bacteria(1.0000),p:Firmicutes(1.0000),c:Clostridia(0.9961),o:Clostridiales(0.9970),f:Lachnospiraceae(0.9878),g:Blautia(0.9743);"	2.321721	1.75E-25	2.61E-24
OTU193	"d:Bacteria(1.0000),p:Firmicutes(0.9998),c:Clostridia(0.9939),o:Clostridiales(0.9947),f:Lachnospiraceae(0.9274),g:Blautia(0.7814);"	1.935297	7.50E-22	1.02E-20
OTU2828	"d:Bacteria(1.0000),p:Firmicutes(0.9984),c:Clostridia(0.9937),o:Clostridiales(0.9926),f:Ruminococcaceae(0.9847),g:Faecalibacterium(0.8158);"	1.901137	1.17E-21	1.46E-20
OTU106	"d:Bacteria(1.0000),p:Firmicutes(0.8620),c:Clostridia(0.6471),o:Clostridiales(0.3394),f:Ruminococcaceae(0.1097),g:Ruminococcus(0.0143);"	-3.72733	2.04E-21	2.33E-20
OTU38	"d:Bacteria(1.0000),p:Firmicutes(0.9984),c:Negativicutes(0.9935),o:Selenomonadales(0.9904),f:Veillonellaceae(0.9867),g:Dialister(0.9701);"	4.160084	3.37E-21	3.58E-20
OTU116	"d:Bacteria(1.0000),p:Firmicutes(1.0000),c:Clostridia(0.9950),o:Clostridiales(0.9960),f:Lachnospiraceae(0.9888),g:Roseburia(0.9786);"	1.988125	1.29E-20	1.28E-19
OTU23	"d:Bacteria(1.0000),p:Bacteroidetes(0.9996),c:Bacteroidia(0.9974),o:Bacteroidales(0.9986),f:Prevotellaceae(0.9687),g:Prevotella(0.9722);"	2.053714	1.32E-19	1.23E-18
OTU21	"d:Bacteria(1.0000),p:Proteobacteria(0.9987),c:Betaproteobacteria(0.9115),o:Rhodocyclales(0.5981),f:Rhodocyclaceae(0.4275),g:Denitratisoma(0.1672);"	2.40201	2.87E-19	2.52E-18
OTU63	"d:Bacteria(1.0000),p:Firmicutes(0.5785),c:Clostridia(0.0888),o:Clostridiales(0.0400),f:Lachnospiraceae(0.0061),g:Lachnospira(0.0043);"	-4.74902	4.54E-19	3.76E-18
OTU50	"d:Bacteria(1.0000),p:Actinobacteria(1.0000),c:Actinobacteria(1.0000),o:Bifidobacteriales(0.9966),f:Bifidobacteriaceae(0.9970),g:Bifidobacterium(0.9871);"	2.961455	1.25E-18	9.82E-18
OTU143	"d:Bacteria(1.0000),p:Firmicutes(0.9994),c:Clostridia(0.9704),o:Clostridiales(0.9597),f:Lachnospiraceae(0.8051),g:Clostridium_XlVa(0.6095);"	1.929327	1.95E-18	1.45E-17
OTU62	"d:Bacteria(1.0000),p:Bacteroidetes(0.9329),c:Bacteroidia(0.8348),o:Bacteroidales(0.4658),f:Prevotellaceae(0.1097),g:Alloprevotella(0.0143);"	-3.69348	7.95E-17	5.64E-16
OTU76	"d:Bacteria(1.0000),p:Firmicutes(0.9996),c:Clostridia(0.9741),o:Clostridiales(0.9672),f:Lachnospiraceae(0.8778),g:Clostridium_XlVa(0.7126);"	1.742319	9.36E-17	6.34E-16
OTU7	"d:Bacteria(1.0000),p:Firmicutes(0.9991),c:Clostridia(0.9964),o:Clostridiales(0.9975),f:Ruminococcaceae(0.9973),g:Faecalibacterium(0.9829);"	1.348277	1.29E-16	8.37E-16
OTU93	"d:Bacteria(1.0000),p:Firmicutes(0.9978),c:Clostridia(0.9299),o:Clostridiales(0.8337),f:Ruminococcaceae(0.5430),g:Sporobacter(0.0865);"	1.648018	1.60E-16	9.96E-16
OTU92	"d:Bacteria(1.0000),p:Firmicutes(0.9993),c:Clostridia(0.9391),o:Clostridiales(0.8477),f:Ruminococcaceae(0.6553),g:Ruminococcus(0.1672);"	-3.15022	3.58E-16	2.08E-15
OTU166	"d:Bacteria(1.0000),p:Firmicutes(1.0000),c:Clostridia(0.9958),o:Clostridiales(0.9968),f:Lachnospiraceae(0.9770),g:Blautia(0.9743);"	1.662982	3.62E-16	2.08E-15
OTU69	"d:Bacteria(1.0000),p:Firmicutes(0.9991),c:Clostridia(0.9668),o:Clostridiales(0.9457),f:Ruminococcaceae(0.4677),g:Butyricicoccus(0.2194);"	2.54981	1.05E-15	5.82E-15
OTU118	"d:Bacteria(1.0000),p:Firmicutes(0.9999),c:Clostridia(0.9990),o:Clostridiales(0.9994),f:Ruminococcaceae(0.9992),g:Subdoligranulum(0.6438);"	1.689002	1.32E-15	7.03E-15
OTU177	"d:Bacteria(1.0000),p:Firmicutes(0.9986),c:Clostridia(0.9931),o:Clostridiales(0.9841),f:Ruminococcaceae(0.9687),g:Gemmiger(0.4282);"	2.374859	3.53E-15	1.81E-14
OTU130	"d:Bacteria(1.0000),p:Firmicutes(0.9984),c:Clostridia(0.9814),o:Clostridiales(0.9715),f:Lachnospiraceae(0.9357),g:Lachnospiracea_incertae_sedis(0.9189);"	1.7531	4.24E-15	2.11E-14
OTU39	"d:Bacteria(1.0000),p:Firmicutes(0.7674),c:Negativicutes(0.2725),o:Selenomonadales(0.1218),f:Veillonellaceae(0.0454),g:Selenomonas(0.0119);"	-2.21457	8.67E-14	4.17E-13
OTU18	"d:Bacteria(1.0000),p:Proteobacteria(0.9329),c:Alphaproteobacteria(0.5845),o:Kiloniellales(0.1942),f:Kiloniellaceae(0.0883),g:Kiloniella(0.0167);"	-3.2637	8.37E-13	3.90E-12
OTU20	"d:Bacteria(1.0000),p:Tenericutes(0.9954),c:Mollicutes(0.9299),o:Anaeroplasmatales(0.7305),f:Anaeroplasmataceae(0.5430),g:Anaeroplasma(0.3238);"	-3.53874	2.33E-12	1.05E-11
OTU67	"d:Bacteria(1.0000),p:Firmicutes(0.6840),c:Clostridia(0.3342),o:Clostridiales(0.1579),f:Lachnospiraceae(0.0296),g:Coprococcus(0.0077);"	-2.0139	3.94E-12	1.73E-11
OTU32	"d:Bacteria(1.0000),p:Tenericutes(0.9970),c:Mollicutes(0.9449),o:Anaeroplasmatales(0.8057),f:Anaeroplasmataceae(0.6179),g:Anaeroplasma(0.4282);"	-3.05316	1.81E-11	7.69E-11
OTU53	"d:Bacteria(1.0000),p:Verrucomicrobia(0.8620),c:Subdivision5(0.6471),g:Subdivision5_genera_incertae_sedis(0.0409);"	2.660803	1.89E-11	7.81E-11
OTU803	"d:Bacteria(1.0000),p:Firmicutes(0.9993),c:Clostridia(0.9522),o:Clostridiales(0.8897),f:Ruminococcaceae(0.6928),g:Oscillibacter(0.1672);"	1.311371	2.12E-11	8.55E-11
OTU66	"d:Bacteria(1.0000),p:Actinobacteria(0.2622),c:Actinobacteria(0.0888),o:Actinomycetales(0.0400),f:Nocardiaceae(0.0140),g:Rhodococcus(0.0060);"	2.341409	5.03E-11	1.97E-10
OTU47	"d:Bacteria(1.0000),p:Firmicutes(0.9566),c:Clostridia(0.8470),o:Clostridiales(0.5099),f:Ruminococcaceae(0.2263),g:Clostridium_IV(0.0167);"	-1.87224	7.02E-11	2.68E-10
OTU3166	"d:Bacteria(1.0000),p:Firmicutes(0.9946),c:Clostridia(0.8654),o:Clostridiales(0.5981),f:Ruminococcaceae(0.2665),g:Sporobacter(0.0523);"	1.131313	1.07E-10	4.00E-10
OTU55	"d:Bacteria(1.0000),p:Firmicutes(0.7911),c:Clostridia(0.4593),o:Clostridiales(0.2305),f:Clostridiaceae_1(0.0668),g:Clostridium_sensu_stricto(0.0143);"	-2.39178	2.27E-10	8.25E-10
OTU111	"d:Bacteria(1.0000),p:Firmicutes(0.9998),c:Clostridia(0.9922),o:Clostridiales(0.9778),f:Lachnospiraceae(0.8943),g:Clostridium_XlVa(0.6095);"	1.273272	5.69E-10	2.02E-09
OTU179	"d:Bacteria(1.0000),p:Firmicutes(0.9962),c:Clostridia(0.9023),o:Clostridiales(0.7746),f:Ruminococcaceae(0.5055),g:Sporobacter(0.0751);"	-1.68821	1.26E-09	4.36E-09
OTU72	"d:Bacteria(1.0000),p:Firmicutes(0.4731),c:Clostridia(0.2113),o:Clostridiales(0.0673),f:Lachnospiraceae(0.0061),g:Lachnospiracea_incertae_sedis(0.0043);"	-1.85938	1.94E-09	6.56E-09
OTU146	"d:Bacteria(1.0000),p:Firmicutes(0.7911),c:Negativicutes(0.2725),o:Selenomonadales(0.1218),f:Veillonellaceae(0.0454),g:Dendrosporobacter(0.0119);"	-1.44193	2.79E-09	9.23E-09
OTU3	"d:Bacteria(1.0000),p:Bacteroidetes(0.9914),c:Bacteroidia(0.6471),o:Bacteroidales(0.3394),f:Porphyromonadaceae(0.1525),g:Paludibacter(0.0215);"	1.637282	3.05E-09	9.88E-09
OTU223	"d:Bacteria(1.0000),p:Firmicutes(0.9993),c:Clostridia(0.9299),o:Clostridiales(0.8337),f:Lachnospiraceae(0.5055),g:Clostridium_XlVa(0.0865);"	1.076341	4.73E-09	1.50E-08

OTU217	"d:Bacteria(1.0000),p:Firmicutes(0.3677),c:Clostridia(0.1500),o:Clostridiales(0.0673),f:Ruminococcaceae(0.0140),g:Ruminococcus(0.0060);"	-2.60026	1.28E-08	3.98E-08
OTU188	"d:Bacteria(1.0000),p:Firmicutes(0.9914),c:Clostridia(0.8746),o:Clostridiales(0.5540),f:Ruminococcaceae(0.2665),g:Intestinimonas(0.0295);"	1.333667	3.27E-08	9.96E-08
OTU36	"d:Bacteria(1.0000),p:Bacteroidetes(0.9995),c:Bacteroidia(0.9973),o:Bacteroidales(0.9985),f:Prevotellaceae(0.9687),g:Prevotella(0.9722);"	1.22626	3.67E-08	1.10E-07
OTU46	"d:Bacteria(1.0000),p:Firmicutes(0.9914),c:Clostridia(0.8562),o:Clostridiales(0.5540),f:Ruminococcaceae(0.3068),g:Pseudobacteroides(0.0295);"	1.02569	9.83E-08	2.87E-07
OTU428	"d:Bacteria(1.0000),p:Firmicutes(0.9899),c:Clostridia(0.7723),o:Clostridiales(0.4216),f:Clostridiaceae_1(0.1311),g:Clostridium_sensu_stricto(0.0215);"	1.138146	2.41E-07	6.91E-07
OTU91	"d:Bacteria(1.0000),p:Firmicutes(0.9946),c:Clostridia(0.5845),o:Clostridiales(0.3031),f:Clostridiaceae_1(0.1525),g:Anaerosporobacter(0.0295);"	-1.18845	3.16E-07	8.89E-07
OTU119	"d:Bacteria(1.0000),p:Firmicutes(0.9990),c:Clostridia(0.9777),o:Clostridiales(0.9693),f:Ruminococcaceae(0.8943),g:Ruminococcus(0.6438);"	-1.74448	5.77E-07	1.59E-06
OTU31	"d:Bacteria(1.0000),p:Bacteroidetes(0.9987),c:Bacteroidia(0.9704),o:Bacteroidales(0.9597),f:Rikenellaceae(0.9109),g:Alistipes(0.7814);"	-1.43744	9.65E-07	2.61E-06
OTU1431	"d:Bacteria(1.0000),p:Firmicutes(0.9994),c:Clostridia(0.9971),o:Clostridiales(0.9983),f:Ruminococcaceae(0.9847),g:Faecalibacterium(0.6782);"	0.968951	1.05E-06	2.78E-06
OTU322	"d:Bacteria(1.0000),p:Firmicutes(0.9990),c:Clostridia(0.9956),o:Clostridiales(0.9966),f:Eubacteriaceae(0.9888),g:Eubacterium(0.9914);"	1.296944	1.52E-06	3.96E-06
OTU164	"d:Bacteria(1.0000),p:Firmicutes(0.9954),c:Clostridia(0.7097),o:Clostridiales(0.3775),f:Lachnospiraceae(0.1861),g:Syntrophococcus(0.0409);"	-1.72156	1.73E-06	4.43E-06
OTU78	"d:Bacteria(1.0000),p:Firmicutes(0.6840),c:Clostridia(0.3342),o:Clostridiales(0.1579),f:Peptostreptococcaceae(0.0454),g:Acetoanaerobium(0.0060);"	-1.8134	1.75E-06	4.43E-06
OTU27	"d:Bacteria(1.0000),p:Bacteroidetes(0.9988),c:Bacteroidia(0.9850),o:Bacteroidales(0.9736),f:Prevotellaceae(0.8861),g:Alloprevotella(0.6782);"	-1.9834	1.90E-06	4.73E-06
OTU90	"d:Bacteria(1.0000),p:Firmicutes(0.9970),c:Clostridia(0.9207),o:Clostridiales(0.8197),f:Eubacteriaceae(0.4275),g:Eubacterium(0.1672);"	1.184428	2.22E-06	5.42E-06
OTU158	"d:Bacteria(0.6762),p:Proteobacteria(0.1203),c:Deltaproteobacteria(0.0195),o:Syntrophobacterales(0.0074),f:Syntrophaceae(0.0020),g:Desulfomonile(0.0020);"	-1.65293	2.25E-06	5.42E-06
OTU270	"d:Bacteria(1.0000),p:Firmicutes(0.8384),c:Clostridia(0.5219),o:Clostridiales(0.2668),f:Ruminococcaceae(0.0296),g:Clostridium_III(0.0094);"	1.020501	2.60E-06	6.15E-06
OTU49	"d:Bacteria(1.0000),p:Firmicutes(0.9978),c:Clostridia(0.9391),o:Clostridiales(0.8477),f:Ruminococcaceae(0.6179),g:Oscillibacter(0.0751);"	0.872358	2.98E-06	6.94E-06
OTU19	"d:Bacteria(1.0000),p:Bacteroidetes(0.9988),c:Bacteroidia(0.8654),o:Bacteroidales(0.5981),f:Porphyromonadaceae(0.4275),g:Parabacteroides(0.0409);"	-1.90432	4.59E-06	1.04E-05
OTU35	"d:Bacteria(1.0000),p:Firmicutes(0.8620),c:Clostridia(0.6471),o:Clostridiales(0.2668),f:Ruminococcaceae(0.1311),g:Intestinimonas(0.0191);"	1.663946	4.61E-06	1.04E-05
OTU43	"d:Bacteria(1.0000),p:Firmicutes(0.8147),c:Clostridia(0.5219),o:Clostridiales(0.2668),f:Ruminococcaceae(0.0668),g:Ruminococcus(0.0094);"	-1.79852	6.84E-06	1.52E-05
OTU41	"d:Bacteria(1.0000),p:Firmicutes(0.7674),c:Clostridia(0.3967),o:Clostridiales(0.1942),f:Ruminococcaceae(0.0218),g:Intestinimonas(0.0077);"	-1.42623	8.29E-06	1.82E-05
OTU2966	"d:Bacteria(1.0000),p:Firmicutes(0.9899),c:Clostridia(0.5845),o:Clostridiales(0.3031),f:Ruminococcaceae(0.1525),g:Pseudobacteroides(0.0215);"	-0.96919	9.64E-06	2.08E-05
OTU131	"d:Bacteria(1.0000),p:Firmicutes(0.9987),c:Clostridia(0.9950),o:Clostridiales(0.9960),f:Ruminococcaceae(0.9919),g:Faecalibacterium(0.9701);"	0.862111	1.04E-05	2.21E-05
OTU4	"d:Bacteria(1.0000),p:Spirochaetes(0.7911),c:Spirochaetia(0.4593),o:Spirochaetales(0.2305),f:Spirochaetaceae(0.1097),g:Spirochaeta(0.0094);"	-1.58297	1.13E-05	2.37E-05
OTU149	"d:Bacteria(1.0000),p:Fibrobacteres(0.9985),c:Fibrobacteria(0.9948),o:Fibrobacterales(0.9958),f:Fibrobacteraceae(0.9929),g:Fibrobacter(0.9968);"	-1.48993	1.40E-05	2.90E-05
OTU40	"d:Bacteria(1.0000),p:Firmicutes(1.0000),c:Clostridia(0.9950),o:Clostridiales(0.9960),f:Lachnospiraceae(0.9888),g:Roseburia(0.9533);"	0.804764	8.85E-05	0.000181
OTU89	"d:Bacteria(1.0000),p:Firmicutes(0.5785),c:Clostridia(0.2725),o:Clostridiales(0.1218),f:Ruminococcaceae(0.0218),g:Ruminococcus(0.0077);"	-0.83959	8.99E-05	0.000181
OTU28	"d:Bacteria(1.0000),p:Proteobacteria(0.8147),c:Alphaproteobacteria(0.3342),o:Kiloniellales(0.0673),f:Kiloniellaceae(0.0218),g:Kiloniella(0.0077);"	1.729008	0.000114	0.000227
OTU73	"d:Bacteria(1.0000),p:Proteobacteria(0.7674),c:Alphaproteobacteria(0.2113),o:Kiloniellales(0.0400),f:Kiloniellaceae(0.0140),g:Kiloniella(0.0060);"	1.424816	0.00012	0.000236
OTU145	"d:Bacteria(1.0000),p:Firmicutes(0.5785),c:Erysipelotrichia(0.2113),o:Erysipelotrichales(0.0945),f:Erysipelotrichaceae(0.0296),g:Erysipelothrix(0.0094);"	1.269654	0.000153	0.000296
OTU99	"d:Bacteria(1.0000),p:Actinobacteria(0.9938),c:Actinobacteria(0.8931),o:Coriobacteriales(0.7305),f:Coriobacteriaceae(0.5430),g:Slackia(0.0751);"	1.069212	0.000184	0.000351
OTU202	"d:Bacteria(1.0000),p:Firmicutes(0.9899),c:Clostridia(0.8348),o:Clostridiales(0.4658),f:Ruminococcaceae(0.2263),g:Clostridium_III(0.0215);"	-0.95072	0.000191	0.00036
OTU1219	"d:Bacteria(1.0000),p:Proteobacteria(0.5785),c:Deltaproteobacteria(0.2725),o:Bdellovibrionales(0.1218),f:Bdellovibrionaceae(0.0454),g:Vampirovibrio(0.0119);"	-1.5597	0.000219	0.000409
OTU835	"d:Bacteria(1.0000),p:Bacteroidetes(0.9914),c:Bacteroidia(0.5845),o:Bacteroidales(0.3031),f:Porphyromonadaceae(0.1311),g:Paludibacter(0.0215);"	-1.73913	0.000363	0.000668
OTU159	"d:Bacteria(0.9753),p:Bacteroidetes(0.6840),c:Sphingobacteriia(0.0285),o:Sphingobacteriales(0.0111),f:Sphingobacteriaceae(0.0037),g:Parapedobacter(0.0031);"	1.301056	0.000558	0.001014
OTU14	"d:Bacteria(1.0000),p:Firmicutes(0.6840),c:Clostridia(0.3342),o:Clostridiales(0.1579),f:Clostridiaceae_4(0.0296),g:Thermotalea(0.0094);"	-1.43384	0.000847	0.00152
OTU115	"d:Bacteria(0.9753),p:Firmicutes(0.1875),c:Clostridia(0.0345),o:Clostridiales(0.0136),f:Clostridiaceae_1(0.0042),g:Caloramator(0.0034);"	-1.22151	0.001081	0.001917
OTU1	"d:Bacteria(1.0000),p:Firmicutes(1.0000),c:Clostridia(0.9958),o:Clostridiales(0.9968),f:Clostridiaceae_1(0.9857),g:Sarcina(0.9722);"	-1.05785	0.001604	0.002812
OTU113	"d:Bacteria(1.0000),p:Bacteroidetes(0.9987),c:Bacteroidia(0.9924),o:Bacteroidales(0.9799),f:Prevotellaceae(0.4275),g:Prevotella(0.0637);"	-1.24938	0.001827	0.003166
OTU107	"d:Bacteria(1.0000),p:Firmicutes(0.9983),c:Clostridia(0.9924),o:Clostridiales(0.9799),f:Lachnospiraceae(0.9191),g:Anaerostipes(0.8846);"	0.920972	0.002201	0.00377
OTU22	"d:Bacteria(1.0000),p:Bacteroidetes(0.9922),c:Bacteroidia(0.8348),o:Bacteroidales(0.4658),f:Prevotellaceae(0.1525),g:Paraprevotella(0.0295);"	-0.91249	0.002274	0.00385
OTU102	"d:Bacteria(1.0000),p:Proteobacteria(0.2622),c:Deltaproteobacteria(0.0888),o:Bdellovibrionales(0.0400),f:Bdellovibrionaceae(0.0140),g:Vampirovibrio(0.0060);"	0.90778	0.002421	0.004053
OTU126	"d:Bacteria(1.0000),p:Firmicutes(0.7911),c:Clostridia(0.4593),o:Clostridiales(0.2305),f:Clostridiaceae_4(0.0296),g:Thermotalea(0.0094);"	0.814249	0.002491	0.004124
OTU900	"d:Bacteria(1.0000),p:Firmicutes(0.9998),c:Clostridia(0.9981),o:Clostridiales(0.9956),f:Ruminococcaceae(0.9898),g:Faecalibacterium(0.8158);"	0.665968	0.002555	0.004184
OTU128	"d:Bacteria(1.0000),p:Bacteroidetes(0.9984),c:Bacteroidia(0.9937),o:Bacteroidales(0.9926),f:Prevotellaceae(0.6553),g:Prevotella(0.3760);"	-1.18638	0.003132	0.005073
OTU3172	"d:Bacteria(1.0000),p:Firmicutes(0.9992),c:Clostridia(0.9926),o:Clostridiales(0.9820),f:Ruminococcaceae(0.9357),g:Faecalibacterium(0.5327);"	0.540922	0.003508	0.00562
OTU45	"d:Bacteria(0.6389),p:Lentisphaerae(0.0530),c:Lentisphaeria(0.0165),o:Victivallales(0.0062),f:Victivallaceae(0.0015),g:Victivallis(0.0017);"	0.938299	0.005721	0.009069

Table S9. OTUs whose relative abundances correlate significantly with the number of chloroplast reads classified as Poaceae.

OUT#	Taxonomy	Correlation	P-value	% Relative abundance
OTU101	d:Bacteria(1.0000),p:Proteobacteria(0.9994),c:Betaproteobacteria(0.9850),o:Burkholderiales(0.9693),f:Sutterellaceae(0.9191),g:Sutterella(0.8846)	0.42	0.010	0.13
OTU224	d:Bacteria(0.2356),p:Proteobacteria(0.0162),c:Alphaproteobacteria(0.0098),o:Rhodospirillales(0.0036),f:Acetobacteraceae(0.0005),g:Stella(0.0009)	0.63	<0.001	0.17
OTU225	d:Bacteria(1.0000),p:Firmicutes(0.9990),c:Clostridia(0.9207),o:Clostridiales(0.8197),f:Lachnospiraceae(0.4677),g:Clostridium_XlVa(0.1672)	0.46	<0.001	0.14
OTU324	d:Bacteria(1.0000),p:Firmicutes(0.9930),c:Clostridia(0.8562),o:Clostridiales(0.5540),f:Clostridiales_Incertae_Sedis_XIII(0.3068),g:Anaerovorax(0.0751)	0.44	0.004	0.02
OTU450	d:Bacteria(1.0000),p:Firmicutes(0.4731),c:Clostridia(0.2113),o:Clostridiales(0.0945),f:Clostridiales_Incertae_Sedis_XII(0.0296),g:Guggenheimella(0.0094)	0.41	0.016	0.04
OTU456	d:Bacteria(1.0000),p:Firmicutes(0.9986),c:Clostridia(0.9850),o:Clostridiales(0.9736),f:Lachnospiraceae(0.8861),g:Roseburia(0.5751)	0.43	0.006	0.08
OTU585	d:Bacteria(1.0000),p:Firmicutes(0.9991),c:Clostridia(0.9559),o:Clostridiales(0.9037),f:Lachnospiraceae(0.6928),g:Clostridium_XlVa(0.3760)	0.42	0.007	0.12
OTU925	d:Bacteria(1.0000),p:Firmicutes(0.9990),c:Clostridia(0.9595),o:Clostridiales(0.9177),f:Lachnospiraceae(0.6553),g:Clostridium_XlVa(0.3238)	0.45	0.002	0.01
OTU1873	d:Bacteria(1.0000),p:Firmicutes(0.9993),c:Clostridia(0.8746),o:Clostridiales(0.6422),f:Lachnospiraceae(0.3470),g:Anaerostipes(0.0865)	0.41	0.020	0.01

Table S10. Sample overview

Species	Population	Habitat type	Season	PCR1 Fwd primer	PCR1 Rev primer	PCR2 Rev primer	# reads
Bale monkey	Afursa	Forest fragment	Wet	f1	r12	r8	176040
Bale monkey	Afursa	Forest fragment	Wet	f1	r15	r8	148340
Bale monkey	Afursa	Forest fragment	Wet	f1	r16	r8	170335
Bale monkey	Afursa	Forest fragment	Wet	f1	r19	r8	55537
Bale monkey	Afursa	Forest fragment	Wet	f1	r22	r8	122032
Bale monkey	Afursa	Forest fragment	Wet	f1	r3	r8	147838
Bale monkey	Afursa	Forest fragment	Wet	f1	r4	r8	169076
Bale monkey	Afursa	Forest fragment	Dry	f1	r7	r8	98070
Bale monkey	Afursa	Forest fragment	Wet	f10	r12	r8	158401
Bale monkey	Afursa	Forest fragment	Dry	f10	r15	r8	154711
Bale monkey	Afursa	Forest fragment	Wet	f10	r16	r8	203879
Bale monkey	Afursa	Forest fragment	Wet	f10	r19	r8	189863
Bale monkey	Afursa	Forest fragment	Dry	f10	r22	r8	144893
Bale monkey	Afursa	Forest fragment	Wet	f10	r3	r8	160245
Bale monkey	Afursa	Forest fragment	Dry	f10	r4	r8	191819
Bale monkey	Afursa	Forest fragment	Dry	f10	r7	r8	210598
Bale monkey	Afursa	Forest fragment	Wet	f2	r12	r8	197771
Bale monkey	Afursa	Forest fragment	Wet	f2	r15	r8	195875
Bale monkey	Afursa	Forest fragment	Wet	f2	r16	r8	197817
Bale monkey	Afursa	Forest fragment	Wet	f2	r19	r8	242609
Bale monkey	Afursa	Forest fragment	Wet	f2	r22	r8	116917
Bale monkey	Afursa	Forest fragment	Wet	f2	r3	r8	99646
Bale monkey	Afursa	Forest fragment	Wet	f2	r4	r8	205900
Bale monkey	Afursa	Forest fragment	Wet	f2	r7	r8	204234
Bale monkey	Afursa	Forest fragment	Wet	f4	r12	r8	99476
Bale monkey	Afursa	Forest fragment	Wet	f4	r15	r8	132708
Bale monkey	Afursa	Forest fragment	Wet	f4	r16	r8	140356
Bale monkey	Afursa	Forest fragment	Wet	f4	r19	r8	92779
Bale monkey	Afursa	Forest fragment	Wet	f4	r22	r8	89497
Bale monkey	Afursa	Forest fragment	Wet	f4	r3	r8	103240
Bale monkey	Afursa	Forest fragment	Wet	f4	r7	r8	90224
Bale monkey	Afursa	Forest fragment	Dry	f5	r12	r8	145189
Bale monkey	Afursa	Forest fragment	Dry	f5	r15	r8	148120
Bale monkey	Afursa	Forest fragment	Wet	f5	r16	r8	183271
Bale monkey	Afursa	Forest fragment	Dry	f5	r19	r8	171777
Bale monkey	Afursa	Forest fragment	Dry	f5	r22	r8	105238
Bale monkey	Afursa	Forest fragment	Wet	f5	r3	r8	162181
Bale monkey	Afursa	Forest fragment	Dry	f5	r4	r8	152297
Bale monkey	Afursa	Forest fragment	Dry	f5	r7	r8	149129
Bale monkey	Afursa	Forest fragment	Wet	f6	r4	r8	186330
Bale monkey	Afursa	Forest fragment	Dry	f6	r7	r8	191101
Bale monkey	Afursa	Forest fragment	Dry	f7	r12	r8	161767
Bale monkey	Afursa	Forest fragment	Dry	f7	r15	r8	159379
Bale monkey	Afursa	Forest fragment	Dry	f7	r16	r8	156692
Bale monkey	Afursa	Forest fragment	Dry	f7	r19	r8	135518
Bale monkey	Afursa	Forest fragment	Dry	f7	r22	r8	156887
Bale monkey	Afursa	Forest fragment	Dry	f7	r3	r8	91829
Bale monkey	Afursa	Forest fragment	Dry	f7	r4	r8	74617
Bale monkey	Afursa	Forest fragment	Dry	f8	r12	r8	194030
Bale monkey	Afursa	Forest fragment	Dry	f8	r16	r8	134240
Bale monkey	Afursa	Forest fragment	Dry	f8	r19	r8	132262
Bale monkey	Afursa	Forest fragment	Dry	f8	r3	r8	87175
Bale monkey	Afursa	Forest fragment	Dry	f8	r4	r8	137499
Bale monkey	Afursa	Forest fragment	Dry	f8	r7	r8	139141
Bale monkey	Afursa	Forest fragment	Dry	f2	r16	r9	60444
Bale monkey	Afursa	Forest fragment	Dry	f2	r19	r9	185696
Bale monkey	Afursa	Forest fragment	Dry	f2	r22	r9	141783
Bale monkey	Afursa	Forest fragment	Dry	f4	r12	r9	85176
Bale monkey	Afursa	Forest fragment	Dry	f4	r15	r9	102107
Bale monkey	Afursa	Forest fragment	Dry	f4	r16	r9	89836
Bale monkey	Afursa	Forest fragment	Dry	f4	r19	r9	112529
Bale monkey	Afursa	Forest fragment	Dry	f4	r22	r9	83428
Bale monkey	Afursa	Forest fragment	Dry	f4	r3	r9	92979
Bale monkey	Afursa	Forest fragment	Dry	f4	r7	r9	89505
Bale monkey	Afursa	Forest fragment	Dry	f5	r3	r9	93889
Bale monkey	Afursa	Forest fragment	Dry	f6	r4	r9	121440
Bale monkey	Kokosa	Forest fragment	Dry	f10	r12	r9	165812

Bale monkey	Kokosa	Forest fragment	Dry	f10	r15	r9	163081
Bale monkey	Kokosa	Forest fragment	Dry	f10	r16	r9	84495
Bale monkey	Kokosa	Forest fragment	Dry	f10	r19	r9	172657
Bale monkey	Kokosa	Forest fragment	Dry	f10	r22	r9	132667
Bale monkey	Kokosa	Forest fragment	Dry	f10	r3	r9	102676
Bale monkey	Kokosa	Forest fragment	Dry	f10	r4	r9	134016
Bale monkey	Kokosa	Forest fragment	Dry	f10	r7	r9	158581
Bale monkey	Kokosa	Forest fragment	Dry	f11	r12	r9	192877
Bale monkey	Kokosa	Forest fragment	Dry	f11	r15	r9	105987
Bale monkey	Kokosa	Forest fragment	Wet	f11	r19	r9	101434
Bale monkey	Kokosa	Forest fragment	Wet	f11	r22	r9	114592
Bale monkey	Kokosa	Forest fragment	Dry	f11	r3	r9	85795
Bale monkey	Kokosa	Forest fragment	Dry	f11	r4	r9	84034
Bale monkey	Kokosa	Forest fragment	Dry	f11	r7	r9	114815
Bale monkey	Kokosa	Forest fragment	Wet	f13	r12	r9	182997
Bale monkey	Kokosa	Forest fragment	Wet	f13	r15	r9	148375
Bale monkey	Kokosa	Forest fragment	Wet	f13	r16	r9	134372
Bale monkey	Kokosa	Forest fragment	Wet	f13	r19	r9	160718
Bale monkey	Kokosa	Forest fragment	Wet	f13	r22	r9	81295
Bale monkey	Kokosa	Forest fragment	Wet	f13	r3	r9	120502
Bale monkey	Kokosa	Forest fragment	Wet	f13	r4	r9	123492
Bale monkey	Kokosa	Forest fragment	Wet	f13	r7	r9	112237
Bale monkey	Kokosa	Forest fragment	Wet	f16	r12	r9	150836
Bale monkey	Kokosa	Forest fragment	Wet	f16	r15	r9	115191
Bale monkey	Kokosa	Forest fragment	Wet	f16	r19	r9	191063
Bale monkey	Kokosa	Forest fragment	Wet	f16	r3	r9	142630
Bale monkey	Kokosa	Forest fragment	Wet	f16	r4	r9	125921
Bale monkey	Kokosa	Forest fragment	Wet	f16	r7	r9	162574
Bale monkey	Kokosa	Forest fragment	Wet	f19	r12	r9	189199
Bale monkey	Kokosa	Forest fragment	Wet	f19	r15	r9	166788
Bale monkey	Kokosa	Forest fragment	Wet	f19	r16	r9	198949
Bale monkey	Kokosa	Forest fragment	Wet	f19	r22	r9	151453
Bale monkey	Kokosa	Forest fragment	Wet	f19	r3	r9	135794
Bale monkey	Kokosa	Forest fragment	Wet	f19	r4	r9	151500
Bale monkey	Kokosa	Forest fragment	Wet	f19	r7	r9	108163
Bale monkey	Kokosa	Forest fragment	Wet	f22	r12	r9	147180
Bale monkey	Kokosa	Forest fragment	Wet	f22	r15	r9	162107
Bale monkey	Kokosa	Forest fragment	Wet	f22	r3	r9	138706
Bale monkey	Kokosa	Forest fragment	Wet	f22	r4	r9	121914
Bale monkey	Kokosa	Forest fragment	Wet	f22	r7	r9	129637
Bale monkey	Kokosa	Forest fragment	Dry	f5	r12	r9	136242
Bale monkey	Kokosa	Forest fragment	Dry	f5	r15	r9	132521
Bale monkey	Kokosa	Forest fragment	Dry	f5	r16	r9	53268
Bale monkey	Kokosa	Forest fragment	Dry	f5	r19	r9	111171
Bale monkey	Kokosa	Forest fragment	Dry	f5	r22	r9	69201
Bale monkey	Kokosa	Forest fragment	Dry	f5	r7	r9	120455
Bale monkey	Kokosa	Forest fragment	Wet	f6	r19	r9	219888
Bale monkey	Kokosa	Forest fragment	Dry	f6	r7	r9	137429
Bale monkey	Kokosa	Forest fragment	Dry	f7	r12	r9	144913
Bale monkey	Kokosa	Forest fragment	Dry	f7	r15	r9	148326
Bale monkey	Kokosa	Forest fragment	Dry	f7	r16	r9	127038
Bale monkey	Kokosa	Forest fragment	Dry	f7	r19	r9	105732
Bale monkey	Kokosa	Forest fragment	Dry	f7	r3	r9	88205
Bale monkey	Kokosa	Forest fragment	Dry	f7	r4	r9	110842
Bale monkey	Kokosa	Forest fragment	Dry	f8	r12	r9	145805
Bale monkey	Kokosa	Forest fragment	Dry	f8	r15	r9	116079
Bale monkey	Kokosa	Forest fragment	Dry	f8	r16	r9	132250
Bale monkey	Kokosa	Forest fragment	Dry	f8	r19	r9	113327
Bale monkey	Kokosa	Forest fragment	Dry	f8	r22	r9	95115
Bale monkey	Kokosa	Forest fragment	Dry	f8	r3	r9	105109
Bale monkey	Kokosa	Forest fragment	Dry	f8	r4	r9	116778
Bale monkey	Kokosa	Forest fragment	Dry	f8	r7	r9	172816
Bale monkey	Odobullu	Contiuous forest	Dry	f11	r12	r8	189589
Bale monkey	Odobullu	Contiuous forest	Dry	f11	r15	r8	80505
Bale monkey	Odobullu	Contiuous forest	Wet	f11	r19	r8	192023
Bale monkey	Odobullu	Contiuous forest	Dry	f11	r4	r8	126655
Bale monkey	Odobullu	Contiuous forest	Dry	f11	r7	r8	142312
Bale monkey	Odobullu	Contiuous forest	Wet	f13	r12	r8	165545
Bale monkey	Odobullu	Contiuous forest	Dry	f13	r15	r8	139670

Bale monkey	Odobullu	Contiuous forest	Wet	f13	r16	r8	159181
Bale monkey	Odobullu	Contiuous forest	Wet	f13	r19	r8	172418
Bale monkey	Odobullu	Contiuous forest	Wet	f13	r22	r8	93905
Bale monkey	Odobullu	Contiuous forest	Wet	f13	r3	r8	150710
Bale monkey	Odobullu	Contiuous forest	Wet	f13	r4	r8	139578
Bale monkey	Odobullu	Contiuous forest	Wet	f13	r7	r8	172701
Bale monkey	Odobullu	Contiuous forest	Wet	f16	r12	r8	149525
Bale monkey	Odobullu	Contiuous forest	Wet	f16	r15	r8	145818
Bale monkey	Odobullu	Contiuous forest	Wet	f16	r19	r8	121922
Bale monkey	Odobullu	Contiuous forest	Dry	f16	r3	r8	126370
Bale monkey	Odobullu	Contiuous forest	Dry	f16	r4	r8	146606
Bale monkey	Odobullu	Contiuous forest	Wet	f16	r7	r8	147274
Bale monkey	Odobullu	Contiuous forest	Dry	f19	r12	r8	159341
Bale monkey	Odobullu	Contiuous forest	Wet	f19	r15	r8	181547
Bale monkey	Odobullu	Contiuous forest	Dry	f19	r16	r8	151276
Bale monkey	Odobullu	Contiuous forest	Dry	f19	r22	r8	65044
Bale monkey	Odobullu	Contiuous forest	Dry	f19	r3	r8	194022
Bale monkey	Odobullu	Contiuous forest	Dry	f19	r4	r8	169760
Bale monkey	Odobullu	Contiuous forest	Dry	f19	r7	r8	158885
Bale monkey	Odobullu	Contiuous forest	Dry	f22	r15	r8	154127
Bale monkey	Odobullu	Contiuous forest	Wet	f6	r16	r8	195226
Bale monkey	Odobullu	Contiuous forest	Dry	f6	r19	r8	176078
Grivet	Asella	Plantation/forest fragment	Wet	f1	r12	r9	83723
Grivet	Asella	Plantation/forest fragment	Wet	f1	r15	r9	133915
Grivet	Asella	Plantation/forest fragment	Wet	f1	r16	r9	159591
Grivet	Asella	Plantation/forest fragment	Wet	f1	r19	r9	102430
Grivet	Asella	Plantation/forest fragment	Wet	f1	r22	r9	147245
Grivet	Asella	Plantation/forest fragment	Wet	f1	r3	r9	87990
Grivet	Asella	Plantation/forest fragment	Wet	f1	r4	r9	79583
Grivet	Asella	Plantation/forest fragment	Wet	f1	r7	r9	135857
Grivet	Asella	Plantation/forest fragment	Wet	f2	r12	r9	134338
Grivet	Asella	Plantation/forest fragment	Wet	f2	r15	r9	124949
Grivet	Asella	Plantation/forest fragment	Wet	f2	r3	r9	131862
Grivet	Asella	Plantation/forest fragment	Wet	f2	r4	r9	155026
Grivet	Awassa	Woodland	Dry	f16	r22	r7	150827
Grivet	Awassa	Woodland	Dry	f19	r12	r7	119614
Grivet	Awassa	Woodland	Dry	f19	r15	r7	204763
Grivet	Awassa	Woodland	Dry	f19	r16	r7	142145
Grivet	Awassa	Woodland	Dry	f19	r22	r7	103867
Grivet	Awassa	Woodland	Dry	f19	r3	r7	166438
Grivet	Awassa	Woodland	Dry	f19	r4	r7	164697
Grivet	Awassa	Woodland	Dry	f19	r7	r7	165174
Grivet	Awassa	Woodland	Dry	f22	r12	r7	119186
Grivet	Awassa	Woodland	Dry	f22	r15	r7	115746
Grivet	Awassa	Woodland	Dry	f22	r3	r7	110701
Grivet	Awassa	Woodland	Dry	f22	r7	r7	108092
Grivet	Awassa	Woodland	Dry	f6	r19	r7	157760
Grivet	Wondo Genet	Forest fragment	Wet	f10	r12	r7	120500
Grivet	Wondo Genet	Forest fragment	Wet	f10	r15	r7	152229
Grivet	Wondo Genet	Forest fragment	Wet	f10	r16	r7	151862
Grivet	Wondo Genet	Forest fragment	Wet	f10	r19	r7	118603
Grivet	Wondo Genet	Forest fragment	Wet	f10	r22	r7	102853
Grivet	Wondo Genet	Forest fragment	Wet	f10	r3	r7	178141
Grivet	Wondo Genet	Forest fragment	Wet	f10	r4	r7	178064
Grivet	Wondo Genet	Forest fragment	Wet	f11	r3	r7	149059
Grivet	Wondo Genet	Forest fragment	Wet	f11	r4	r7	155180
Grivet	Wondo Genet	Forest fragment	Wet	f8	r12	r7	151632
Grivet	Wondo Genet	Forest fragment	Wet	f8	r15	r7	156107
Grivet	Wondo Genet	Forest fragment	Wet	f8	r16	r7	150317
Grivet	Wondo Genet	Forest fragment	Wet	f8	r19	r7	143729
Grivet	Wondo Genet	Forest fragment	Wet	f8	r22	r7	116192
Vervet	Sof Omar	Tree dominated forest	Wet	f11	r12	r7	125912
Vervet	Sof Omar	Tree dominated forest	Wet	f11	r15	r7	128308
Vervet	Sof Omar	Tree dominated forest	Wet	f11	r19	r7	183132
Vervet	Sof Omar	Tree dominated forest	Wet	f11	r7	r7	155061
Vervet	Sof Omar	Tree dominated forest	Wet	f13	r12	r7	136301
Vervet	Sof Omar	Tree dominated forest	Wet	f13	r15	r7	162349
Vervet	Sof Omar	Tree dominated forest	Wet	f13	r16	r7	148777
Vervet	Sof Omar	Tree dominated forest	Wet	f13	r19	r7	85703

Vervet	Sof Omar	Tree dominated forest	Wet	f13	r22	r7	140301
Vervet	Sof Omar	Tree dominated forest	Wet	f13	r4	r7	133540
Vervet	Sof Omar	Tree dominated forest	Wet	f13	r7	r7	135196
Vervet/grivet	Arba Minch	Plantation/natural forest	Dry	f6	r7	r7	78532
Vervet/grivet	Arba Minch	Plantation/natural forest	Dry	f7	r12	r7	51015
Vervet/grivet	Arba Minch	Plantation/natural forest	Dry	f7	r15	r7	68608
Vervet/grivet	Arba Minch	Plantation/natural forest	Dry	f7	r16	r7	160061
Vervet/grivet	Arba Minch	Plantation/natural forest	Dry	f7	r19	r7	96297
Vervet/grivet	Arba Minch	Plantation/natural forest	Dry	f7	r22	r7	122043
Vervet/grivet	Arba Minch	Plantation/natural forest	Dry	f7	r3	r7	76847
Vervet/grivet	Arba Minch	Plantation/natural forest	Dry	f7	r4	r7	154700
Vervet/grivet	Arba Minch	Plantation/natural forest	Dry	f8	r3	r7	70823
Vervet/grivet	Arba Minch	Plantation/natural forest	Dry	f8	r4	r7	131442
Vervet/grivet	Arba Minch	Plantation/natural forest	Dry	f8	r7	r7	155585
